# CRISPR Element Patterns vs. Pathoadaptability of Clinical *Pseudomonas aeruginosa* Isolates from a Medical Center in Moscow, Russia

**DOI:** 10.3390/antibiotics10111301

**Published:** 2021-10-26

**Authors:** Marina Tyumentseva, Yulia Mikhaylova, Anna Prelovskaya, Konstantin Karbyshev, Aleksandr Tyumentsev, Lyudmila Petrova, Anna Mironova, Mikhail Zamyatin, Andrey Shelenkov, Vasiliy Akimkin

**Affiliations:** 1 Central Research Institute of Epidemiology, Novogireevskaya Str., 3a, 111123 Moscow, Russia; tyumentseva@cmd.su (M.T.); mihailova@cmd.su (Y.M.); prelovskaya@cmd.su (A.P.); karbyshev@cmd.su (K.K.); tymencev@cmd.su (A.T.); vgakimkin@yandex.ru (V.A.); 2 National Medical and Surgical Center Named after N.I. Pirogov, Nizhnyaya Pervomayskaya Str., 70, 105203 Moscow, Russia; lutix85@yandex.ru (L.P.); annamir_88@mail.ru (A.M.); mnz1@yandex.ru (M.Z.)

**Keywords:** *Pseudomonas aeruginosa*, WGS, antibiotic resistance, virulence factors, multiple CRISPR/Cas systems, pathoadaptability

## Abstract

*Pseudomonas aeruginosa* is a member of the ESKAPE opportunistic pathogen group, which includes six species of the most dangerous microbes. This pathogen is characterized by the rapid acquisition of antimicrobial resistance, thus causing major healthcare concerns. This study presents a comprehensive analysis of clinical *P. aeruginosa* isolates based on whole-genome sequencing data. The isolate collection studied was characterized by a variety of clonal lineages with a domination of high-risk epidemic clones and different CRISPR/Cas element patterns. This is the first report on the coexistence of two and even three different types of CRISPR/Cas systems simultaneously in Russian clinical strains of *P. aeruginosa*. The data include molecular typing and genotypic antibiotic resistance determination, as well as the phylogenetic analysis of the full-length *cas* gene and anti-CRISPR genes sequences, predicted prophage sequences, and conducted a detailed CRISPR array analysis. The differences between the isolates carrying different types and quantities of CRISPR/Cas systems were investigated. The pattern of virulence factors in *P. aeruginosa* isolates lacking putative CRISPR/Cas systems significantly differed from that of samples with single or multiple putative CRISPR/Cas systems. We found significant correlations between the numbers of prophage sequences, antibiotic resistance genes, and virulence genes in *P. aeruginosa* isolates with different patterns of CRISPR/Cas-elements. We believe that the data presented will contribute to further investigations in the field of bacterial pathoadaptability, including antimicrobial resistance and the role of CRISPR/Cas systems in the plasticity of the *P. aeruginosa* genome.

## 1. Introduction

*Pseudomonas aeruginosa*, a Gram-negative opportunistic human pathogen, is a one of the most common bacteria worldwide that causes nosocomial infections, including sepsis, pneumonia and urinary tract infections (UTI; see Abbreviations for a full list of the abbreviations used in the manuscript). Compared to other pathogens, *P. aeruginosa* is very difficult to eradicate as it displays a high intrinsic resistance to a wide variety of antibiotics, including aminoglycosides, fluoroquinolones and β-lactams [1]. Most of the strains are able to enhance their resistance by the acquisition of resistance determinants using horizontal gene transfer, which results in the emergence of multidrug-resistant (MDR), or even pan drug-resistant, strains of *P. aeruginosa*. Due to this capacity, it was included in the group of “superbugs” and added to the WHO’s Global List of critical, priority-level strains that require scientific research and the development of new antibiotics [2].

The population of *P. aeruginosa* is characterized by an epidemic structure, i.e., frequent recombination, the conservation of the common gene pool in strains from various sources and the spreading of epidemic clones characterized by the conservation of structural genomic regions [3]. The global population of *P. aeruginosa* is heterogeneous and consists of a large number of diverse genotypes. Based on the multilocus sequence typing (MLST) data, several high-risk epidemic clones were defined: ST 111, 175, 233, 235, 277, 357, 654 and 773 [4,5,6,7]. *P. aeruginosa* strains belonging to these groups are more often characterized by MDR or an extreme resistance phenotype due to the acquisition of various antibiotic resistance (AR) genes, including metallo-β-lactamases (MBL-genes). According to the MLST-typing of the Russian *P. aeruginosa* population, carbapenemase-producing strains exhibited a rather limited variety of genotypes belonging to several clonal complexes: CC235, CC654, CC111, CC244, and CC313, with first two complexes being predominant [4]. Another mechanism conferring the carbapenem resistance in Russian *P. aeruginosa* isolates is the inactivation of porin channels, primarily the OprD porin, which could be achieved by alterations in the *oprD* gene sequence [8].

In comparison to other bacterial pathogens, *P. aeruginosa* has a large genome (5.5–7 Mb), and it harbors many horizontally transferable elements, such as plasmids, conjugative elements, prophages, pathogenicity islands, integrons, and transposons forming an accessory genome of the pathogen [9]. CRISPR/Cas systems are widely distributed among *P. aeruginosa* strains. About 50% of the sequenced *P. aeruginosa* genomes were predicted to possess an active CRISPR/Cas system, which also played an important role in shaping the accessory genomes of the globally distributed strains [10]. Notably, *P. aeruginosa* was used as a bacterial model system for studying the molecular mechanisms of Type I CRISPR/Cas system functioning [11]. Three major CRISPR/Cas system types (I-F, I-E and I-C) were identified in *P. aeruginosa*, wherein the first type was the most frequent, while the I-C-type was rather rare [12]. In a large-scale study, Van Belkum et al. [12] analyzed the CRISPR/Cas systems of 672 *P. aeruginosa* strains and correlated their presence or absence with antibiotic resistance. They showed that CRISPR/Cas systems in the genomes studied contributed to phage resistance, rather than hampering the horizontal transfer of beneficial mobile genetic elements, including resistance genes. However, there are some works on *Acinetobacter baumannii*, *Enterococcus faecalis*, *Streptococcus pneumoniae* and *Staphylococcus aureus* that show the direct involvement of CRISPR/Cas systems in the interruption of the horizontal gene transfer [13,14,15]. The analysis of correlations between the CRISPR/Cas, anti-CRISPR, and antibiotic resistance gene content of more than 100,000 reference genomes of 5677 different species, including *P. aeruginosa*, allowed hypothesizing that the selection for antibiotic resistance could result in an accumulation of anti-CRISPR genes in the genomes that harbored both CRISPR/Cas systems and antibiotic resistance genes acquired by horizontal transfer [10].

The analysis of the CRISPR arrays in Brazilian *P. aeruginosa* isolates harboring I-F type systems showed that CRISPR loci exhibited a high plasticity and spacer diversity, but, at the same time, similarities between the strains and spacer rearrangements were observed [16]. These authors also reported the presence of I-F and I-E types in the same isolate simultaneously. In a later work, the same research group characterized 13 Brazilian isolates harboring CRISPR/Cas systems, their phage protein and anti-CRISPR protein contents, and their genetic relationships with other publicly available genomes. This study revealed that similar CRISPR/Cas types and spacer contents were present in the strains with the same ST, and that I-F/I-E strains and lineages were not closely related [17].

In the present study, we provide comprehensive data based on the whole genome sequencing for 51 clinical isolates of *P. aeruginosa* obtained from a multidisciplinary medical center in Moscow (referenced as ‘CriePir’ isolates below). All the isolates, except one, carried CRISPR arrays and different types of CRISPR/Cas systems. A significant part of the population studied was characterized by the carriage of two or even three CRISPR/Cas system types presented in the same isolate simultaneously. A comparative analysis allowed the determination of the phylogenetic relationship between the genes encoding the anti-CRISPR and Cas proteins from the isolates studied and from the collection of publicly available global *P. aeruginosa* strains (designated as ‘reference’ strains below). Multiparametric and correlation analyses were conducted in terms of antimicrobial resistance and virulence genomic determinants, as well as other pathoadaptable factors of the isolates under investigation.

## 2. Results

### 2.1. Typing and CRISPR Element Distribution of the P. aeruginosa Isolates

The results of the isolate typing using MLST and lipooligosaccharide outer-core, loci (OCL)-based, in silico serotyping are presented in Table S1. The isolates belonged to twenty different sequence types, according to the PasteurMLST scheme, and were combined into 16 clonal complexes (CC). ST654 was the most abundant (19 samples, 37%); ten isolates were characterized by ST235 (19.6%). These sequence types are the main STs in the respective CCs, which belong to epidemic, high-risk clones associated with multiple and extreme resistances to antimicrobial drugs. The most frequent O-type was O4 (19 isolates in total and all of them belonged to ST 654). All ST235 isolates possessed O11, which was also common for other minor sequence types: ST1076, ST357, ST2592 and ST1567 (Table S1).

The isolate CriePir317 possessed novel ST, which had the following allelic profile: acsA (17), aroE (6), guaA (12), mutL (3), nuoD (14), ppsA (4), trpE (9). We have submitted this isolate to the Institut Pasteur MLST system (http://bigsdb.pasteur.fr, accessed on 9 September 2020), and the database curators assigned a new profile, ST3452, to this allele combination.

It was also interesting that three pairs of isolates (CriePir118/CriePir156, CriePir203/CriePir207, and CriePir235/CriePir287) were obtained from three patients (A17, A31, and A32, respectively), but the isolates in each pair had different STs, which indicated a simultaneous infection by multiple strains. The other two isolates (CriePir153/CriePir161) with ST654 were also collected from different loci of the same patient (A21) but had varied genomic profiles.

Approximately 50% of the *P. aeruginosa* genomes available in public databases are predicted to harbor active CRISPR/Cas systems [12]. Along with the diversity and plasticity of *P. aeruginosa* genomes, this pathogen is characterized by a high variability of CRISPR/Cas systems. Here, we performed, to the best of our knowledge, the first detailed and focused description of CRISPR/Cas elements in Russian *P. aeruginosa* isolates of clinical origin (listed in Table S1), in comparison to the strains of the same species that were available from public databases (listed in Table S2).

**Figure 1:**
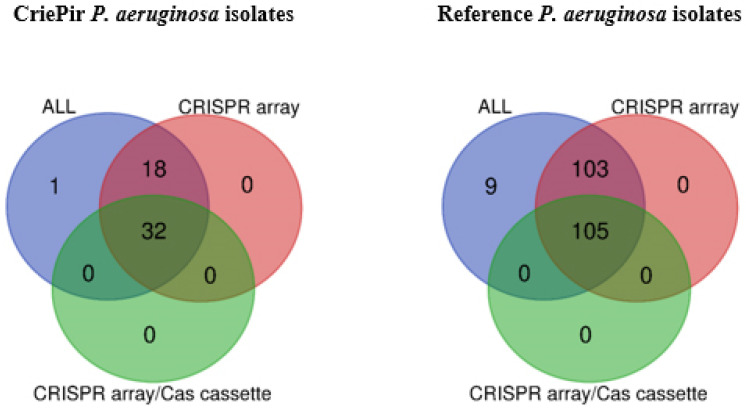
Venn diagrams of CRISPR/Cas element distribution for CriePir *P. aeruginosa* isolates sequenced by us (**left**) and reference isolates available from Pseudomonas genome DB (**right**).

Eighteen isolates (35%) from the set sequenced by us possessed confirmed CRISPR sequences (CRISPR arrays) (Table S1, Figure 1, left). Thirty-two samples (63%) carried different putative CRISPR/Cas systems. Only the isolate CriePir198 from our population did not have either a CRISPR array or putative CRISPR/Cas system.

A similar distribution of CRISPR/Cas elements was observed for the whole genomes in 217 reference *P. aeruginosa* isolates available in the Pseudomonas Genome database (https://www.pseudomonas.com/, accessed on 21 March 2021) [18]. Namely, 103 and 105 isolates were characterized as carrying CRISPR arrays and putative CRISPR/Cas systems, respectively, while nine strains did not have any CRISPR/Cas elements (Figure 1, right). Ten out of eighteen CriePir isolates with CRISPR arrays belonged to ST235.

**Figure 2:**
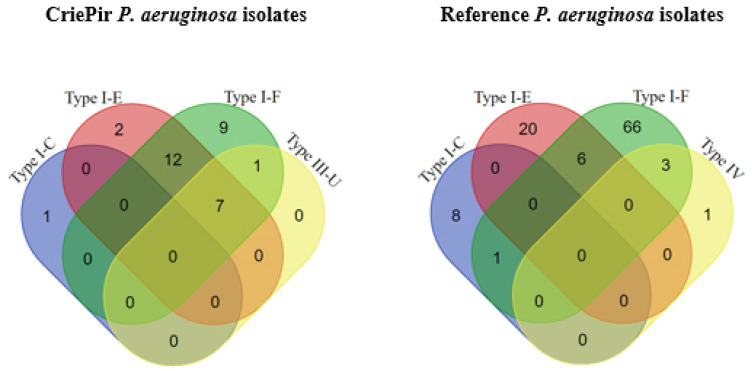
Venn diagrams of CRISPR/Cas system type distribution for CriePir (**left**) and reference (**right**) *P. aeruginosa* isolates.

The distribution of different putative CRISPR/Cas systems containing both CRISPR arrays and cas cassettes in *P. aeruginosa* isolates was investigated (Figure 2). It was shown that putative CRISPR/Cas systems with one cas cassette occurred much more frequently (*p* < 0.00001) in the reference *P. aeruginosa* isolates (Table S2), while CRISPR/Cas systems with two cas cassettes were more frequently (*p* = 0.00001) found in our CriePir *P. aeruginosa* isolates. Namely, two isolates (CriePir171 and CriePir199) carried the CRISPR/Cas Type I-E, while nine CriePir samples were characterized by the CRISPR/Cas system Type I-F. The isolate CriePir118 has a CRISPR/Cas system Type I-C, which is considered to be rarely occurring. Our data are in agreement with the work of van Belkum et al. [12], in which the three mentioned CRISPR/Cas system types (I-F, I-E, and I-C) were already been reported in *P. aeruginosa* strains, with varying frequencies for each type. Type I-F was the most frequent type, and Type I-C was rarely found [12]. The rest of our clinical isolates possessed two or three different CRISPR/Cas systems simultaneously.

The I-E and I-F types were carried by twelve isolates, and the isolate CriePir207 belonging to ST132 and harbored the I-F and III-U types (Table S1, Figure 2, left). Seven isolates possessed three types of CRISPR/Cas systems (I-E, I-F and III-U). Interestingly, all isolates harboring multiple CRISPR/Cas systems (two or three types) belonged to ST654, with the only exception being CriePir207, as mentioned above (Table S1, Figure 2, left). Surprisingly, putative CRISPR/Cas systems with three cas cassettes were found only in our studied *P. aeruginosa* collection (*p* ˂ 0.05).

The CRISPR/Cas loci of the Type I-F isolates were similar and consisted of six genes encoding Cas6 endoribonuclease, three Csy proteins (Csy3, Csy2 and Csy1), Cas3/Cas2 helicase/RNase, and Cas1 endonuclease in the vicinity of CRISPR arrays, except for the CriePir35 and CriePir39 isolates that carried an internal deletion resulting in the absence of Cas6.

Almost all of the Type I-E isolates consisted of seven genes encoding Cas1, Cas2, Cas5, Cas6, Cas7, Cse1, and Cse2. The CriePir35 and CriePir1, Type I-E isolates carried eight cas genes with an additional one coding for Cas3.

The CriePir118 isolate belonged to the Type I-C CRISPR/Cas system and contained four genes encoding the Cas1, Cas5c, Cas7c, and Cas8c proteins.

The CRISPR/Cas loci of all Type III-U containing isolates carried one gene encoding the Csx3 protein. The Csx3 family of Cas proteins is encoded in a CRISPR-associated gene cluster near CRISPR, which is repeated in the genomes of several different thermophiles such as *Archaeoglobus fulgidus*, *Aquifex aeolicus*, *Dictyoglomus thermophilum*, thermophilic Synechococcus, etc. (see Table S3 for the complete list). Csx3 is a distant member of the CARF (CRISPR-associated Rossmann fold) domain superfamily previously characterized as a Mn2+-dependent deadenylation exoribonuclease with phosphodiesterase activity. This strain binds the cyclic tetra-adenylate (cA4) second messenger with a high affinity and quickly degrades the cA4 signal [19]. On the one hand, according to the InterPro resource (http://www.ebi.ac.uk/interpro/entry/InterPro/IPR013409/, accessed on 21 March 2021), the Csx3-containing CRISPR/Cas systems are determined as «Not yet assigned to a specific CRISPR/Cas subtype». On the other hand, Csx3 is strongly associated with Type III systems [19].

It should be noted that none of the genes of the described CRISPR/Cas loci contained premature stop codons.

**Figure 3:**
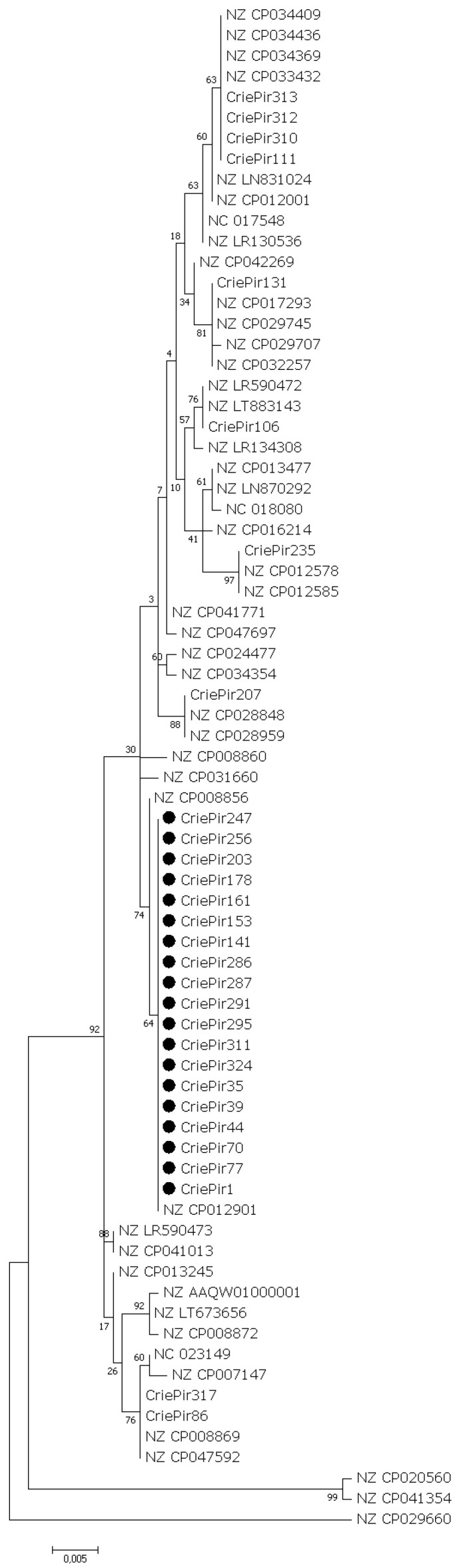
Maximum likelihood phylogenetic tree of full-length csy3 gene sequences of clinical CriePir *P. aeruginosa* isolates and reference isolates from Pseudomonas genome database. Bootstrap test (1000 replicates) was applied, and bootstrap values are shown at the branch nodes. The ST654 sequences identified in this study are marked with black circle. Sequences identified in this study are indicated by the isolate names and the reference sequences, by GenBank accession number.

The maximum-likelihood phylogenetic trees of the full-length cas gene sequences showed that *P. aeruginosa* CriePir ST654 isolates always formed separate clades on phylogenetic trees. CriePir ST2592 and ST357 isolates also formed separate clades, while the topology of the CriePir isolates belonging to the other STs differed slightly between the phylogenetic trees (Figure 3).

To assess the characteristics of *P. aeruginosa,* the CRISPR/Cas system and its role in *P. aeruginosa* pathoadaptibility, all isolates under investigation were divided into five groups according to the CRISPR/Cas system presence: “No CRISPR/No Cas”, “CRISPR/No Cas”, “CRISPR/Single Cas”, “CRISPR/Double Cas”, and “CRISPR/Triple Cas”. To reveal the correlation between the carriage of pathoadaptability factors of *P. aeruginosa* and the CRISPR/Cas system, our isolates were characterized by genotypic antibiotic resistance profiles, plasmid number, presence of genes encoding virulence factors and the number of ambiguous and active prophages.

### 2.2. Antimicrobial Resistance Genotypes vs. CRISPR Patterns

We examined the distribution of the known antibiotic resistance genes among the clinical isolates that we sequenced. The results of the resistance determinant annotation are presented in Table S4. Based on the available data, thirty-five isolates (68%) were MDR.

The spectrum of 46 identified resistance determinants included gene clusters providing resistance to aminoglycosides, beta-lactams, chloramphenicol, fluorquinolones, fosfomycin, sulfonamides, and tetracycline. The WGS-identified aph(3)-Ib in most, but not all, of the isolates tested, the intrinsic genes, blaPAO and catB7, were common for all isolates. The genomes tested were characterized by a different set of aminoglycoside cluster genes, but each isolate carried at least one of the genes from this cluster. The FosA gene (fosfomycin resistance) was observed in all isolates except for CriePir156. The data obtained also demonstrated that 39% (20/51) of the genomes were positive for floR and tetG genes (phenicol and tetracyclin resistance, respectively), and sul1 (sulphonamide resistance) was found in 59% (30/51) of the isolates. Some of the isolates (9/51) carried dfrA5, dfrB2 or dfrB5 genes (trimethoprim resistance).

Twenty-five isolates were positive for the blaOXA-396 gene, fourteen for the blaOXA488 gene, and nineteen isolates for the blaVIM-2 gene of metallo-beta-lactamase (MBL). Notably, all of the isolates harboring the blaVIM-2 gene belonged to ST654, while two isolates of the ST235 carried this carbapenemase. The carriage of the other beta-lactamase genes, including blaCARB, blaGES, blaDIM, blaIMP-1 and blaVIM-11, was also revealed in separate isolates. All of the strains studied were characterized by the combined possessing (from two to six) of different types of beta-lactamase genes. The isolate CriePir111 was the one harboring six different beta-lactamase genes (blaOXA-10, blaOXA-2, blaOXA-50, blaPAO, blaVEB and blaVIM-11).

CriePir24 and CriePir27 of ST234 were characterized by the similar profiles of antimicrobial resistance determinants. These isolates also belonged to the O12 serotype, which was associated with multidrug resistance and described in many European, Mediterranean countries, as well as the UK [20]. The only isolate, from the “No CRISPR/No Cas” group, CriePir198, carried a minimum number of antimicrobial resistance determinants (see Tables S1 and S4).

We did not observe any relationship (correlation) between the number of antibiotic resistance genes and CRISPR-elements patterns for the CriePir isolates studied (Figure S1).

### 2.3. Plasmids

The *P. aeruginosa* genomes contain a large repertoire of mobile genetic elements, including phages, transposons, integrative and conjugative elements, and plasmids [21]. Since plasmids play a key role in acquiring antimicrobial drug resistance, we searched the plasmid sequences in the WGS data. The list of plasmids identified in the isolates is shown in Table S5.

Plasmid sequences were found in 17/51 (33%) isolates analyzed. Comparatively, plasmids rarely occur in *P. aeruginosa* isolates [22]. Most of the plasmid sequences belonged to varieties of Col-type plasmids. Two isolates, CriePir27 and CriePir97, carried plasmids IncQ1 and IncQ2, respectively. Another two isolates, CriePir39 and CriePir274, were characterized as harboring five different Col-type plasmid sequences in each genome. To obtain reliable whole-plasmid sequences, the long-read sequencing could be used, which could also provide additional information regarding plasmid typing and gene content. There was no correlation between the presence/absence or number of plasmids with the CRISPR element patterns (see Figure S1).

### 2.4. Virulence Genes

Bacterial virulence is an important contributor to infectious diseases and complicates treatments with the currently available antibiotics. *P. aeruginosa* has many virulence factors, for example, toxins, proteases and lipases, as well as the factors providing cytotoxicity, biofilm formation, and swarming motility [1]. We identified the virulence factors in the clinical isolates investigated by a bioinformatics analysis of WGS data. The complete list of detected virulence genes is shown in Table S6. An extensive spectrum of 246 identified virulence genes included 44 clusters determining the structural components of the cell, metabolic proteins, flagellar complex, chemotaxis, and secreted products such as exotoxins, etc. The MDR isolate, CriePir156, carried a minimum number of virulence factors (177 genes in total), while the maximum number of virulence determinants was 234 genes harbored by three MDR isolates: CriePir1, CriePir35, and CriePir256. The most common virulence genes were those found in all the studied isolates and represented the following clusters: alg, apr, chp, dot, exo, exs, fle, flg, flh, fli, fpt, hcp, hsi, las, lip, mbth-like, mot, muc, pch, pcr, phz, pil, plc, pop, ppp, psc, pvd, rhl, tag, tse, vgr, waa, and xcp. Seventeen isolates carried the ExoU gene (Table S6), which was the major virulence factor responsible for alveolar epithelial injury in patients with *P. aeruginosa* pneumonia. In *P. aeruginosa* clinical isolates, the exoU+ genotype correlated with a stronger virulence as well as fluoroquinolone and carbapenem resistance phenotypes [23].

The number of virulence factors did not differ between the analyzed CRISPR element groups of the isolates (Figure S1, section C). However, significant differences were found in the frequencies of occurrence of the virulence factor genes: exoS, exoU, fimT, fimU, fleI/flag, fleP, flgL, fliC, fliD, fliS, pilC, pilE, pilV, pilW, pilX, pilY1, pilY2, spcU, and toxA in *P. aeruginosa* isolates with different CRISPR/Cas systems (Table S7).

ExoS is a bifunctional Type III Secretion System virulence factor that rearranges the actin cytoskeleton and induces apoptosis in the target host cells [24]. It occurred more frequently in “CRISPR/Double Cas” and “CRISPR/Triple Cas” and was found in all *P. aeruginosa* isolates belonging to these groups, which could be arranged in descending order: “CRISPR/Triple Cas”, “CRISPR/Double Cas” > “CRISPR/Single Cas” > “CRISPR/No Cas”.

ExoU is a potent (powerful) cytotoxin with phospholipase A2 activity capable of inducing rapid, necrotic cytotoxicity in various eukaryotic cells [24]. ExoU and spcU (specific Pseudomonas chaperone for ExoU [25]) were overrepresented in the “CRISPR/No Cas” and “CRISPR/Single Cas” groups (“CRISPR/No Cas” > “CRISPR/Single Cas” > “CRISPR/Double Cas”, “CRISPR/Triple Cas”). Interestingly, the genes encoding ExoU and SpcU were completely absent in the “CRISPR/Double Cas” and “CRISPR/Triple Cas” groups of *P. aeruginosa* isolates.

The virulence factors, FimT, FimU, PilV, PilW, PilX, PilY1, PilY2, and PilE, involved in Type IV pili (T4P system) biosynthesis, which play a role in a wide variety of functions, including deoxyribonucleic acid uptake, surface attachment, biofilm formation and twitching motility [26], were slightly underrepresented only in the “CRISPR/Single Cas” group (significant difference in “CRISPR/Single Cas” < “CRISPR/Double Cas” *p* = 0.0391).

The PilC, which represented another essential inner membrane component of the T4P system and controlled both pilus assembly and disassembly [26], was more frequently found in the “CRISPR/Double Cas” and “CRISPR/Triple Cas” groups.

The flagellar genes fleI/flaG, fleP, flgL, fliC, fliD, and fliS, associated with the swimming, twitching, and swarming motility of bacteria [27], appeared less frequently in “CRISPR/No Cas” and “CRISPR/Single Cas” than in the “CRISPR/Double Cas” and “CRISPR/Triple Cas” groups of *P. aeruginosa* isolates.

ToxA, a virulence gene responsible for the synthesis of exotoxin A, the major member of the type 2 secretion system, is specific to *P. aeruginosa* isolates from diabetic foot and wound infections [28,29,30]. The ToxA toxin plays an important role in impeding wound healing and inflammatory reactions [31,32] and is associated with multidrug resistance in *P. aeruginosa* [33,34]. We found that the toxA gene occurred more frequently in the “CRISPR/Single Cas” than in the “CRISPR/Triple Cas” group (*p* = 0.0449), and the analyzed groups could be arranged in descending order as “CRISPR/Single Cas” > “CRISPR/No Cas” > “CRISPR/Double Cas” > “CRISPR/Triple Cas” for this gene.

### 2.5. Anti-CRISPR Genes

It is known that 24 different families of anti-CRISPR proteins are capable of blocking different CRISPR/Cas systems, and have been identified in viruses, bacterial and archaeal genomes [35,36]. These proteins were first identified in *P. aeruginosa* [37]. It was reported that around a half of the *P. aeruginosa* genome assemblies included anti-CRISPR (Acr) genes (46%; 924 of 2021) [10].

Nineteen isolates from our study carried anti-CRISPR gene sequences. The list of the anti-CRISPR genes that were found is presented in Table 1.

Twelve of the isolates with anti-CRISPR genes were characterized as possessing CRISPR arrays only. The remaining seven isolates were characterized by the presence of anti-CRISPR genes and putative CRISPR/Cas systems, including the isolates harboring two or three different systems simultaneously (CriePir207; CriePir178 and CriePir311, respectively). The anti-CRISPR gene acrIF3 was found in 16 CriePir isolates.

To assess the peculiarities of *P. aeruginosa* anti-CRISPR elements and the characteristics of *P. aeruginosa* CRISPR/Cas systems, a list of 217 *P. aeruginosa* reference isolates (Table S2) was analyzed along with 19 CriePir samples. The phylogenetic analysis of the anti-CRISPR AcrIF3 gene showed that the isolates CriePir178, 10, 177, 311, 27 and 24 formed separate clades on the tree, as well as CriePir174, 156, 249, 274 and 318 (Figure S2). Notably, the last group of isolates was characterized as harboring CRISPR-arrays only, while two CriePir members (178 and 311) of another separate clade carried three different CRISPR/Cas systems simultaneously (Table 1).

Additional phylogenetic trees for the acrIE3, acrIF2, AcrIF3, AcrIF4, and AcrIIC2 genes are presented in Figure S2.

**Figure 4:**
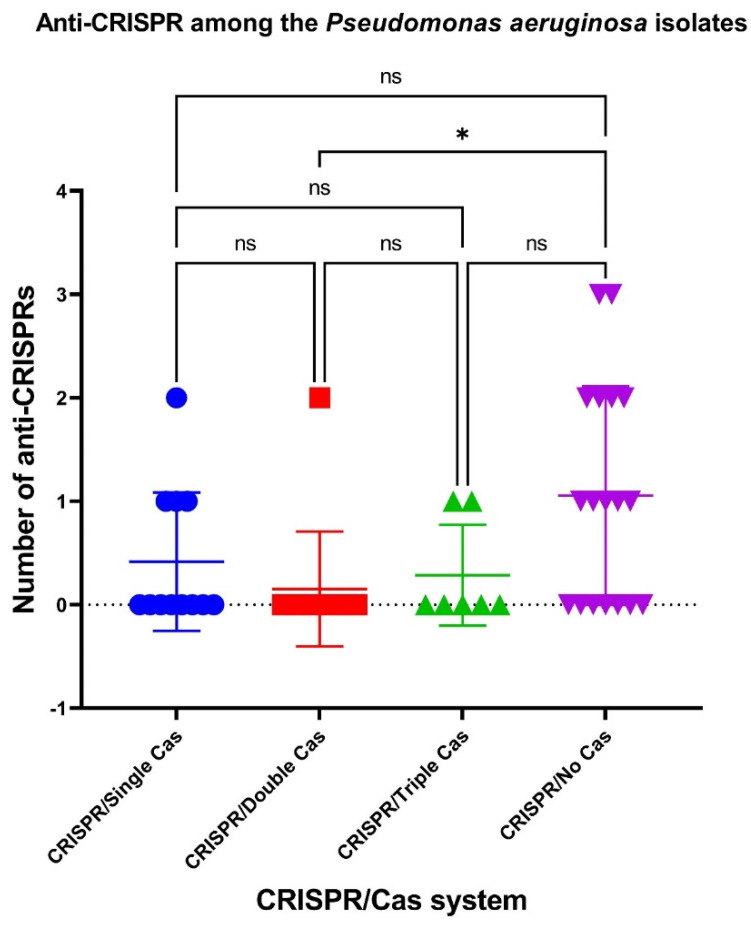
Anti-CRISPRs among the clinical CriePir *P. aeruginosa* isolates from a Moscow medical center with different CRISPR/Cas systems. * indicates the level of significance *p* < 0.05.

It was shown that the number of anti-CRISPRs was significantly (*p* ˂ 0.05) higher in *P. aeruginosa* isolates without cas cassettes (“CRISPR/No Cas” group) than in “CRISPR/Double Cas” isolates (Figure 4).

**Figure 5:**
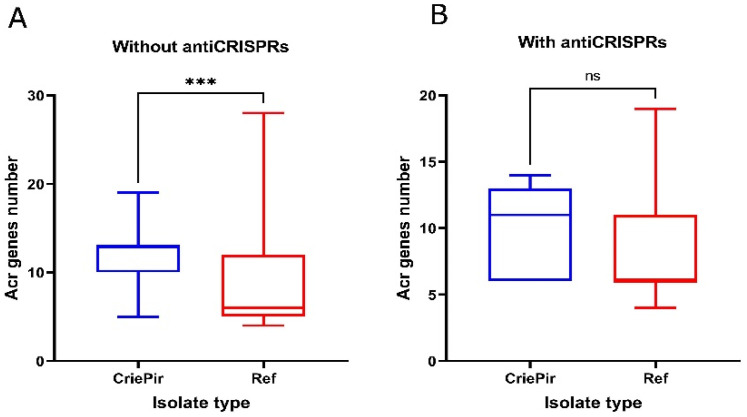

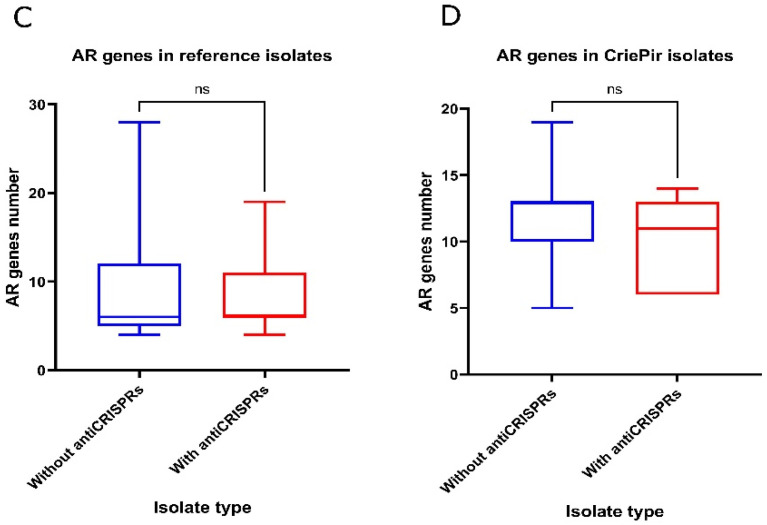
Comparison of the number of AR genes in CriePir and reference (Ref) *P. aeruginosa* isolates with and without antiCRISPRs. The numbers are given for CriePir vs. reference isolates with antiCRISPRs (**A**) and without antiCRISPRs (**B**); with vs. without antiCRISPRs for reference isolates (**C**) and CriePir isolates (**D**); *** indicates the level of significance *p* < 0.001.

The large-scale research by Shehreen et al. [10] showed that, in *P. aeruginosa*, the presence of anti-CRISPRs was associated with antibiotic resistance genes. The authors suggested that anti-CRISPR proteins tended to coexist with AR genes in antibiotic-resistant bacteria by reducing the ability of CRISPR/Cas systems to prevent the acquisition of AR genes. In our study, we assessed the number of AR genes in CriePir and the reference isolates with and without the antiCRISPR genes (Figure 5).

According to the results obtained, the *P. aeruginosa* CriePir isolate collection differed from the reference sample set by a noticeably higher number of AR genes, regardless of the presence or absence of the anti-CRISPR genes (Figure 5A,B), whereas the differences within each group were negligible (Figure 5C,D).

### 2.6. CRISPR Array Types and Prophages

**Figure 6:**
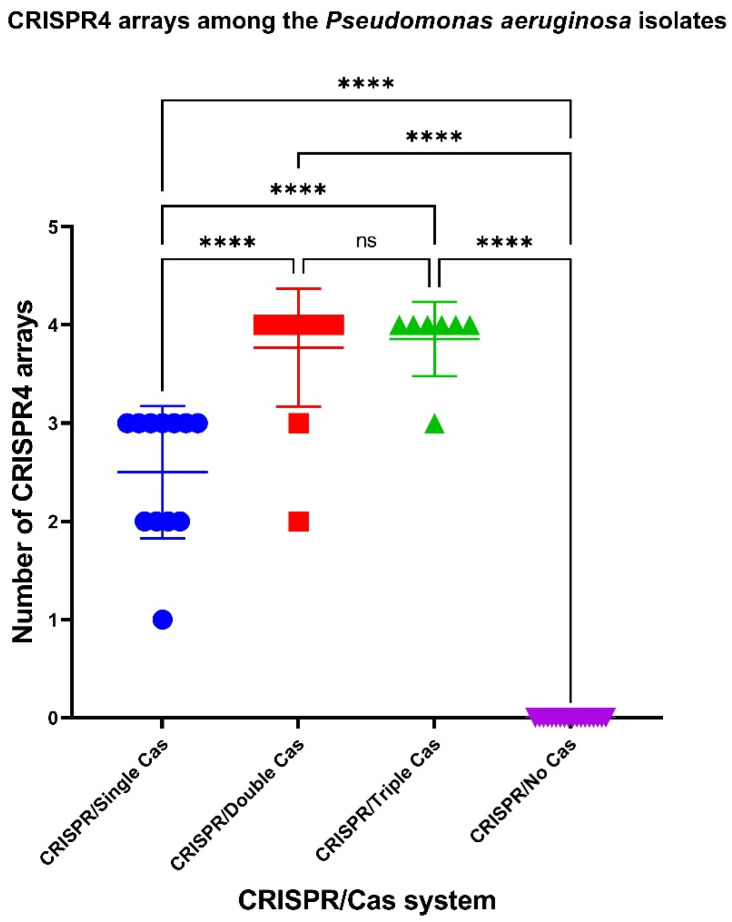
Comparison of CriePir *P. aeruginosa* isolates having different CRISPR/Cas systems by the number of CRISPR4 arrays. **** indicates the level of significance *p* < 0.0001.

To assess the characteristics of the putative CRISPR/Cas systems of *P. aeruginosa*, containing both CRISPR arrays and cas genes, the isolates under investigation were further characterized by CRISPR array types. It was shown that CriePir *P. aeruginosa* isolates carrying CRISPR/Cas systems with multiple cas cassettes had significantly more CRISPR4 arrays (the most reliably predicted CRISPRs) [38] (Figure 6).

It should be noted that no significant differences in the low-evidence CRISPR array numbers were detected in the isolates bearing either “CRISPR/No Cas”, “CRISPR/Single Cas”, “CRISPR/Double Cas”, or “CRISPR/Triple Cas” (Figure S3).

Moreover, no significant differences were seen when comparing the number of active and ambiguous prophages between groups of CriePir *P. aeruginosa* isolates having either “CRISPR/No Cas”, “CRISPR/Single Cas”, “CRISPR/Double Cas”, or “CRISPR/Triple Cas” (Figure S4). This was also true for the number of active prophages and the number of ambiguous prophages among the *P. aeruginosa* isolates belonging to “CRISPR/Single Cas”, “CRISPR/Double Cas” or “CRISPR/Triple Cas” groups (Figure S5).

**Figure 7:**
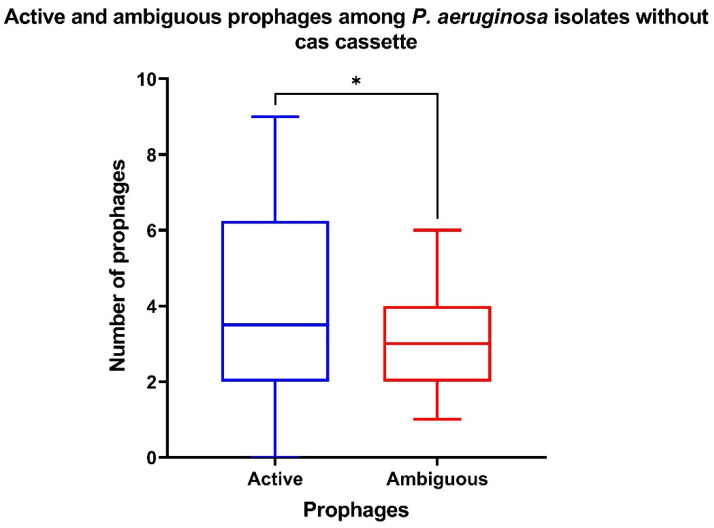
The number of active and ambiguous prophages among CriePir *P. aeruginosa* isolates having no cas cassettes. * indicates the level of significance *p* < 0.05.

At the same time, it was shown that the number of active prophages was significantly (*p* ˂ 0.05) higher than the number of ambiguous prophages in the CriePir samples without cas cassettes (“CRISPR/No Cas” group) (Figure 7).

### 2.7. Correlation Analysis

Additionally, we conducted a correlation analysis. The normalization of the following datasets: “Antibiotic resistance genes”, “Virulence genes” “Plasmids”, “CRISPR”, “CRISPR4” and “Anti-CRISPR” for “CRISPR/No Cas”, “CRISPR/Single Cas”, “CRISPR/Double Cas” and “CRISPR/Triple Cas” groups of CriePir *P. aeruginosa* isolates, was performed, and the nonparametric Spearman correlation was calculated; then, correlation matrices for each pair of datasets were constructed. All the correlation patterns observed for the CriePir isolate groups mentioned above are presented in Figure S6.

For the “CRISPR/No Cas” group of *P. aeruginosa* isolates a significant (*p* < 0.05) negative correlation between the number of antibiotic resistance genes and virulence genes was found (Table 2, Figure S6). Moreover, the number of antibiotic resistance genes positively correlated with the number of active prophages, while a significant (*p* < 0.05) negative correlation was detected between the number of active prophages and the virulence genes (Table 2, Figure S6).

In the “CRISPR/Single Cas” group of *P. aeruginosa* isolates, a significant negative correlation was observed between the number of active prophages and the number of virulence genes, as well as between the number of anti-CRISPR proteins and the number of CRISPR4 arrays. Moreover, a significant positive correlation was seen between the number of antibiotic resistance genes and both the numbers of active prophages and low evidence CRISPR arrays in this group of isolates (Table 2, Figure S6).

In the “CRISPR/Double Cas” group of CriePir, *P. aeruginosa* samples the number of antibiotic resistance genes positively correlated (*p* < 0.05) with the number of virulence genes. At the same time, a significant negative correlation (*p* < 0.05) between the number of ambiguous prophages and the number of active prophages, as well as between the number of low-evidence CRISPR arrays and the number of virulence genes, was demonstrated for the “CRISPR/Double Cas” group of *P. aeruginosa* isolates (Table 2, Figure S6).

A significant positive correlation (*p* < 0.05) was found between the number of antibiotic resistance genes and the number of virulence genes in the “CRISPR/Triple Cas” group of *P. aeruginosa* isolates. Moreover, the number of ambiguous prophages negatively correlated (*p* < 0.05) with both numbers of active prophages and the anti-CRISPR proteins in this group of *P. aeruginosa* isolates (Table 2, Figure S6).

**Figure 8:**
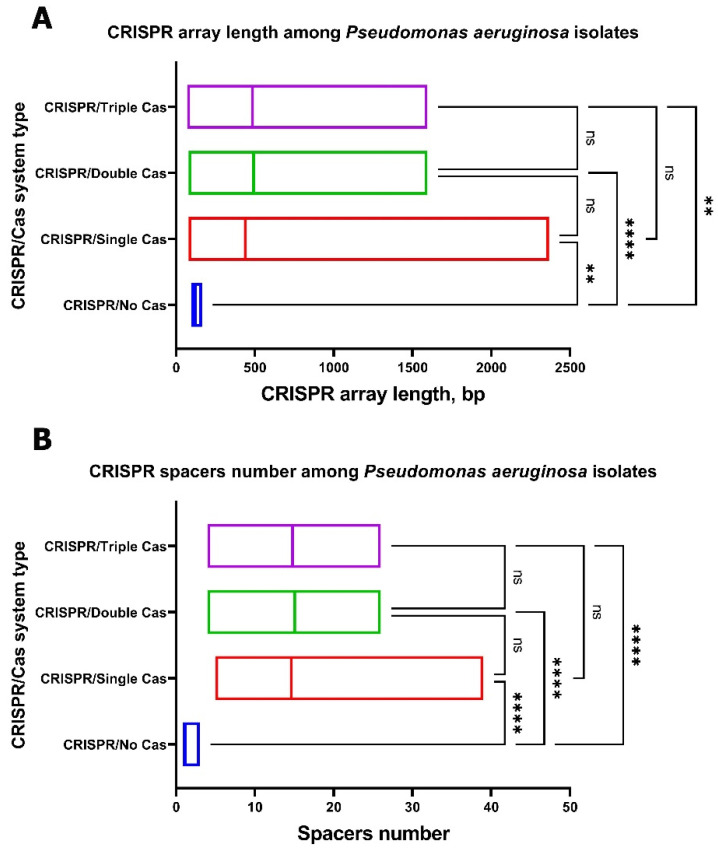
CRISPR array length (**A**) and spacers count (**B**) among CriePir *P. aeruginosa* isolates. ** and **** indicate the increasing levels of significance *p* < 0.01 and *p* < 0.0001, respectively.

Significant differences in the CRISPR array length and the number of spacers were seen between the “CRISPR/Single Cas”, “CRISPR/Double Cas”, and “CRISPR/Triple Cas” groups as well as the “CRISPR/No Cas” group of *P. aeruginosa* isolates (Figure 8). Moreover, significant differences in the CRISPR array length and number of spacers were seen between the “CRISPR/Type I-F” and “CRISPR/Type I-E” groups of *P. aeruginosa* isolates (Figure S7).

The analysis of CRISPR arrays in CriePir *P. aeruginosa* isolates with putative CRISPR/Cas systems revealed 1562 spacers, 204 of which were unique and 1358 were repeating (with 248 unique spacers among the repeating ones). The vast majority (1135 of 1562) of spacers were identified by BLAST (using the MegaBLAST algorithm) as *P. aeruginosa* CRISPR spacers (e.g., ‘*Pseudomonas aeruginosa* strain SMC4518 CRISPR repeat sequence’). Thiry-six percent of spacers were identified by BLAST (using the MegaBLAST algorithm) as phage sequences (e.g., ‘Pseudomonas phage phi297, complete genome’), 12.6% of spacers–as plasmid sequences (e.g., ‘Proteus mirabilis strain L90-1 plasmid pL902, complete sequence’), and 3.5% of spacers as pathogenicity island encoding sequences (e.g., ‘*Pseudomonas aeruginosa* strain CC971 crpP-like-encoding pathogenicity genomic island genomic sequence’) (Table S8). It should be noted that the spacers directed against pathogenicity island-encoding sequences were much more often found in CRISPR arrays of CriePir *P. aeruginosa* isolates harboring multiple CRISPR/Cas systems (two or three types). The possible origins of the spacers are summarized in Figure S8.

Interestingly, eleven spacers (five from the CriePir118 CRISPR array, five from the CriePir311 CRISPR array, and one from the CriePir171 CRISPR array) were not identified by BLAST using the MegaBLAST algorithm (e.g., ‘No significant similarity found’). These spacers were assigned to the Corynebacterium geronticis, Pseudomonas fluorescens, Pseudomonas putida, Pseudomonas sp. CMR5c, Pseudomonas phages, *P. aeruginosa*, Herbaspirillum seropedicae, Burkholderia cenocepacia, Dyadobacter sp. genomes and other bacterial genomes by using the blastn algorithm (Table S8). Two hundred spacers targeted *P. aeruginosa* genomes (Table S8); 53 of the spacers were unique and 147 of them were repeating (with 28 unique spacers among the repeating ones). Three spacers targeted *P. aeruginosa* pyocins (Table S8).

Notably, the CRISPR arrays located upstream and downstream of Type I-E cas cassettes in ST654 CriePir *P. aeruginosa* isolates were identical, except for four spacers of the isolate CriePir311, designated by the MegaBLAST algorithm as ‘No significant similarity found’, and one spacer of CriePir178 (a duplicate of the previous spacer). Three upstream and six downstream spacers of the Type I-E cas cassettes of CriePir171 and CriePir199 isolates were identical.

Twelve upstream spacers, and all downstream spacers (except for two additional spacers of CriePir317), of the Type I-F cas cassettes in the CriePir317 and CriePir86 isolates were identical. The CRISPR arrays located upstream and downstream of Type I-F cas cassettes in CriePir111, 310, 312 and 313 *P. aeruginosa* isolates were also identical. Moreover, the CRISPR arrays located upstream and downstream from the Type I-F cas cassettes in the vast majority of ST654 *P. aeruginosa* isolates were identical, except for one additional spacer targeting the *P. aeruginosa* genome sequence at the end of the CRISPR array, located downstream in CriePir247, 295, 287, 291, 286, 203, 161, 77, 70, 256, 1, and 153. However, ST654 CriePir35 and CriePir39 isolates shared a homology only with the CRISPR arrays located downstream from the Type I-F cas cassettes of the isolates mentioned above.

## 3. Discussion

In this study, we analyzed 51 clinical isolates of *P. aeruginosa* from a Moscow multidisciplinary medical center. MLST is commonly used as the method of choice for the epidemiological surveillance of pathogenic bacteria, if such a scheme exists for the species under study. Additional typing tools may include the determination of the capsule synthesis loci (K-loci)-based strain types (e.g., for *A. baumannii* [39] and *Klebsiella pneumoniae* [40]) or may be performed by using the lipooligosaccharide outer-core loci (OCL) [41]. The specific structure and composition of the O-antigens for *P. aeruginosa* is the basis of classifying the bacteria into O-serotypes. Currently, there are 20 known O-specific antigen structures for *P. aeruginosa* [42]. The auxiliary classification patterns may involve the properties of genomic sequences (e.g., periodicity [43]).

The analyzed samples belonged to 20 different MLST-based sequence types, including the novel ST3452 represented by the isolate CriePir317, and to eight different O-serotypes. Ten isolates of our collection represented the epidemic line of high-risk CC235; the rapid spread and long-term dominance of this strain was observed in Russia [4]. Currently, the hospital epidemiology of infections, which are thought to be caused by *P. aeruginosa,* are characterized by the spread of the new CC654 clone along with the continued dominance of CC235 [44]. This fact is confirmed by our sample set.

CRISPRs were described for a wide range of prokaryotes, but only 36% of bacteria carry both CRISPR arrays and cas genes [38]. According to CRISPRCasdb, about 22% of *Pseudomonas* genus representatives and 45% of isolates from the *P. aeruginosa* species carry both CRISPR arrays and cas genes. These data are consistent with literature where from 36% to 50% of *P. aeruginosa* genomes were considered to possess an active CRISPR/Cas system [10,11,12,22]. In our study, about 63% of CriePir *P. aeruginosa* isolates possessed putative CRISPR/Cas systems. This level of CRISPR/Cas system presence is typical for microbes that produce lactic acid, which are prevalent in both starter cultures and probiotics (CRISPR/Cas systems occur in 62.9% of Lactobacilli genomes and 77% of Bifidobacteria genomes) [45,46,47]. CRISPR/Cas systems are essential for these microbes as they represent the only component for defending against bacteriophages [48], thus representing an essential mechanism of adaptation and survival. Presumably, pathogenic *P. aeruginosa* isolates also use CRISPR/Cas systems for these purposes [49].

Notably, the isolates of ST235 were characterized by harboring CRISPR-arrays only, while ST654 samples carried two or even three different putative CRISPR/Cas systems simultaneously (12 and 7 isolates, respectively). The possession of three CRISPR/Cas systems simultaneously (I-F + I-E + III-U), as well as the combination of I-E and I-F CRISPR/Cas types, were specific characteristics of the ST654 isolates in our *P. aeruginosa* collection. The simultaneous presence of I-E and I-F CRISPR/Cas types was previously documented in several works on single isolates belonging to the STs 252, 387 and 3137 [12,16,17,50]. To the best of our knowledge, this is the first reported case of carrying three different CRISPR/Cas systems simultaneously in the genomes of clinical *P. aeruginosa* isolates. We have not revealed such a property in the 217 reference isolates analyzed.

One isolate in our *P. aeruginosa* collection (CriePir118) harbored a very rare I-C-type CRISPR/Cas system. It is noteworthy that the Type I-C circulated in Brazilian ST277 strains [51], while our isolate belonged to ST399.

Interestingly, the CRISPR/Cas Type III-U that was still «Not yet assigned to a specific CRISPR/Cas subtype» was found in eight CriePir isolates only in combination with two (I-F and I-E) types or one (I-F) other type of isolate (seven ST654 isolates and one ST132 isolate, respectively). None of 217 reference isolates harbored the III-U type CRISPR/Cas system.

Apparently, there are several factors that can contribute to *P. aeruginosa* pathoadaptability, such as the number of antibiotic resistance genes, virulence factors, plasmids, the number of ambiguous and active prophages, and the presence of putative CRISPR/Cas systems. In the present study, we demonstrated that the number of antibiotic resistance genes, plasmid number and the number of genes encoding virulence factors did not differ between the *P. aeruginosa* isolates with different types of CRISPR/Cas systems. In *P. aeruginosa* “CRISPR/No Cas” isolates, several virulence factors responsible for pilus assembly and disassembly [52], as well as the swimming, twitching and swarming motility for bacteria [27,53,54] were underrepresented in comparison to the isolates with putative CRISPR/Cas systems. Some of these factors were associated with poor outcomes in groups of keratitis patients [55] and are known to induce inflammasome and impair bacterial clearance [56], thus making them beneficial in terms of bacterial dissemination. Notably, only ExoU and its specific chaperone, SpcU, were significantly overrepresented among *P. aeruginosa* “CRISPR/No Cas” isolates, while the isolates with multiple putative CRISPR/Cas systems did not have them at all. It is known that ExoU has the greatest impact on disease severity, being associated with severe acute lung injury, sepsis, and mortality. Thus, its prevalence seems not to be beneficial for *P. aeruginosa* dissemination in humans in clinical settings [57,58,59].

Additionally, we found significant correlations between the number of antibiotic resistance genes and the number of virulence genes in *P. aeruginosa* isolates with different types of CRISPR/Cas systems. In the analyzed *P. aeruginosa* isolates, which lacked putative CRISPR/Cas systems, the correlation was negative, while it was positive in the isolates with multiple putative CRISPR/Cas systems. We revealed the pattern of “more virulence genes-less antibiotic resistance genes” and vice versa for the isolates lacking putative CRISPR/Cas systems. However, in the isolates with multiple putative CRISPR/Cas systems we noted the opposite pattern: “more virulence genes-more antibiotic resistance genes”. This fact may also contribute to pathoadaptability of *P. aeruginosa* with multiple putative CRISPR/Cas systems.

Moreover, we showed that *P. aeruginosa* “CRISPR/No Cas” isolates are characterized by the presence of a higher number of active prophage sequences. On the one hand, this fact is consistent with the previously published data [22] and can be linked with the weakness of their “immune” CRISPR/Cas system, i.e., these isolates include less CRISPR4 arrays and possess no cas cassettes, and thus they are not capable to fight against phage infections via CRISPR/Cas. On the other hand, the acquisition of prophage can improve *Pseudomonas* metabolism and increase competitive ability [60,61]. In addition, “CRISPR/No Cas” isolates had more predicted Acr proteins, which could cause the loss of CRISPR/Cas system effectiveness even for the CRISPR/Cas system with cas cassettes acquired from other bacteria [37,62,63,64].

According to the CRISPRminer self-targeting data (http://www.microbiome-bigdata.com/CRISPRminer/index.php/Home/Index/selfTarget, accessed on 21 March 2021), 2240 of the 22,110 self-targeting spacers belonged to *P. aeruginosa*. In our study, the spacers targeting the *P. aeruginosa* genomes were also observed. On the one hand, it is known that CRISPR/Cas systems can acquire self-targeting spacers from the host chromosome, which result in autoimmunity and cell death [65] but, on the other hand, such spacers are suggested to be involved in mRNA degradation that allows the evasion of immune detection [66]. CRISPR/Cas systems possessing self-targeting spacers may require tight regulation to properly balance the danger of autoimmunity with the risk of phage infection, and thus they require further investigations [67].

Interestingly, in our study spacers targeting *P. aeruginosa*, pyocins were found. The pyocin genes are located on the *P. aeruginosa* chromosome and their activities are inducible by mutagenic agents [68]. Pyocins might ensure the predominance of a given strain in a bacterial niche: (i) against other bacteria of the same species, but most *P. aeruginosa* strains are pyocinogenic, (ii) or against other species [68]. Pyocin-targeting spacers, similar to other self-targeting spacers, may be involved in the regulation of the *P. aeruginosa* life cycle.

Taken together, the *P. aeruginosa* isolates with putative CRISPR/Cas systems seem to possess a higher adaptive potential, as they were characterized not only by presence of the so-called prokaryotic “immune system” capable of fighting against phage infections, preventing horizontal gene transfer (e.g., plasmids, PAI, etc.) and providing autoregulation through self-targeting, but also by the arsenal of virulence factors which may contribute to preventing phagocytosis, biofilm establishment, motility, etc. [69,70].

## 4. Materials and Methods

### 4.1. DNA Isolation, Sequencing and Genome Assembly

Fifty-one samples were obtained from 47 patients (34 males and 13 females) from various sources and clinical departments (Table S1) of a multidisciplinary medical center in Moscow, Russia during the period of 2017–2020. The age of the patients involved in this study ranged from 22 to 91 years with a median equal to 63. Initially, the isolates were selected randomly from the set of available samples (125), and some genomes were filtered out later due to insufficient genome coverage and missing metadata, etc.

The optical density at 600 nm (OD_600_) of the media was measured to quantify the microbial growth on agar plates. Genomic DNA was isolated with DNeasy Blood and Tissue kit (Qiagen, Hilden, Germany) and used for paired-end library preparation with Nextera™ DNA Sample Prep Kit (Illumina^®^, San Diego, CA, USA). Nextera™ DNA Sample Prep Kit (Illumina^®^, San Diego, CA, USA) was used for paired-end library preparation, and whole-genome sequencing (WGS) of the isolates on Illumina^®^ Miseq and Hiseq platforms (Illumina^®^, San Diego, CA, USA). Assemblies were obtained using SPAdes versions 3.11, 3.12 and 3.13. All 51 genome assemblies were uploaded to NCBI Genbank under the project number PRJNA744936.

### 4.2. Data Processing

The genomes assembled were processed using custom software pipeline, described earlier [40,71]. We also analyzed 217 reference isolates from Pseudomonas Genome database (the list is presented in Table S2). Our pipeline included the following analyses. For all isolates, we determined the antibiotic resistance genes in silico, performed isolate typing using various molecular classification schemes, and revealed the presence of CRISPR/Cas systems and CRISPR arrays. The presence of virulence factors was also studied. We used Resfinder 4.0 database with default parameters for antimicrobial gene identification (https://cge.cbs.dtu.dk/services/ResFinder/, accessed on 20 March 2021). Plasmid sequences were revealed and typed using PlasmidFinder with default parameters (https://cge.cbs.dtu.dk/services/PlasmidFinder/, accessed on 20 March 2021). CRISPRCasFinder with default parameters [72] was used to identify the presence of CRISPR/Cas systems and spacers in the genomes analyzed.

Venn diagrams were constructed using free webservice (https://bioinformatics.psb.ugent.be/webtools/Venn/, accessed on 20 March 2021). Lists of elements “ALL” (i.e., all isolates), “CRISPR array” (i.e., isolates with predicted CRISPR array) and “CRISPR array/Cas cassette” (i.e., isolates possessing both CRISPR array and Cas cassette) were used for plotting the Venn diagram of CRISPR/Cas elements distribution. Lists with “Type I-C”, “Type I-E”, “Type I-F”, “Type III-U”, and “Type IV” were used in building the Venn diagram reflecting the distribution CRISPR/Cas system type. Each list was inserted into the appropriate fields of the webpage and submitted for analysis. Symmetric graphical output was chosen. To do this, “Symmetric” button was chosen in “OUTPUT control” section for “Venn Diagram Shape”, and “Colored” button for Venn Diagram Fill. The resulting Venn diagrams were exported to PNG format.

Phylogenetic analyses were conducted using Maximum Likelihood (ML) method in MEGA7.0.26 [73]. Briefly, sequences encoding Cas proteins in CriePir and reference isolates were aligned using ClustalW algorithm (“Align DNA” option was used). For pairwise and multiple alignments “Gap Opening Penalty” was set to 15 and “Gap Extension Penalty” to 6.66. The default scoring IUB matrix was used for the comparison of nucleic acid sequences and “Transition Weight” was set to 0.5. Negative Matrix was not used for alignment; “Delay Divergent Cutoff” was set to 30%. Resulting alignments were exported to MEGA format for further analysis. Phylogenetic trees were constructed using Maximum Likelihood (ML) method. The statistical significance of the branches was assessed by bootstrap resampling analysis (1000 replicates).

The presence of anti-CRISPR gene sequences was determined using AcrBank database (http://guolab.whu.edu.cn/anti-CRISPRdb/, accessed on 20 March 2021).

Prophage sequences prediction and evaluation of the probability of a prophage being active were made using Prophage Hunter (https://pro-hunter.genomics.cn/index.php/Home/Index/index.html, accessed on 20 March 2021) [74]. Assembled genomes of CriePir *P. aeruginosa* isolates were uploaded to Prophage Hunter in FASTA format, and prophage sequences were identified using default parameters. The results were downloaded and “01. Main_output” file was analyzed. Prophage sequences belonging to categories “Active” and “Ambiguous” (having scores 0.8–1.0 and 0.5–0.8, respectively) were counted for each CriePir *P. aeruginosa* isolate.

The analysis of spacers in CRISPR arrays of CriePir *P. aeruginosa* isolates with putative CRISPR/Cas systems was performed by Web BLAST^®^. CriePir *P. aeruginosa* spacers were identified and downloaded from CRISPRFinder web tool [75] (http://crispr.i2bc.paris-saclay.fr/Server/, accessed on 20 March 2021). FASTA sequences of CriePir *P. aeruginosa* spacers were uploaded to Web BLAST^®^ blastn suite (https://blast.ncbi.nlm.nih.gov, accessed on 20 March 2021) and analyzed using default parameters of MegaBLAST algorithm.

In order to identify similar CRISPR arrays of CriePir *P. aeruginosa* isolates, sequence alignment by ClustalW algorithm was performed in MEGA7.0.26 [73].

CRISPR array type was assessed using CRISPRCasdb, where CRISPR4 represented Level 4 CRISPRs (the most reliable ones), while CRISPR levels 1, 2 and 3 may be considered as false CRISPRs [38]. Data analysis and graphing were performed using Prism 9 (GraphPad Software, SanDiego, CA, USA).

## 5. Conclusions

Summarizing the results of the research performed, here we presented an assay of a clinical Russian *P. aeruginosa* population consisting of 51 clinical isolates collected from a multidisciplinary medical center during the period of 2017–2020; the majority of isolates possessed a large number of antimicrobial resistance determinants. A detailed analysis of the CRISPR/Cas element patterns, with respect to other pathoadaptability factors (antibiotic resistance genes, virulence determinants, plasmids, and the number of ambiguous and active prophages) allowed us to reveal the specific features of the dominating genetic lines (ST654 and ST235), the isolates harboring CRISPR arrays, and different types of putative CRISPR/Cas systems. Significant correlations between the number of antibiotic resistance genes and the number of virulence genes in *P. aeruginosa* isolates with different types of CRISPR/Cas systems were observed.

The clinical *P. aeruginosa* isolates harboring rare (Type I-C) and multiple (three types) CRISPR/Cas systems simultaneously are of particular interest for our future research. Further experiments on the activity determination of these CRISPR/Cas types and their combinations in pathogenic bacteria are currently underway.

In conclusion, the data obtained can facilitate further investigations in the field of studying the metabolic flexibility, pathoadaptability, and genomic and phenotypic adaptations which promote bacterial survival through understanding the relations between *P. aeruginosa* genotype/phenotypes, the MDR/XDR profile (antibiotic resistance and virulence) and the role of CRISPR/Cas systems in gene transfer and the precise adjustment of *P. aeruginosa* metabolic activities. The results of such investigations, in turn, can facilitate the development of better treatment and prevention strategies for this important pathogen.

## Figures and Tables

**Table 1:** List of the anti-CRISPR genes found in clinical CriePir *P. aeruginosa* isolates from a Moscow multidisciplinary medical center.

Sample ID	CRISPR	Anti-CRISPR Genes
CriePir10	CRISPR array	AcrIF2, AcrIF3
CriePir24	CRISPR array	AcrIF3
CriePir27	CRISPR array	AcrIF3
CriePir97	CRISPR array	AcrIF3, AcrIF4
CriePir106	CRISPR-Cas I-F	AcrIF3,
CriePir118	CRISPR-Cas I-C	AcrIF24, AcrIF23
CriePir156	CRISPR array	AcrIF3
CriePir166	CRISPR array	AcrIF24, AcrIF23, AcrIF4
CriePir171	CRISPR-Cas I-E	AcrIF3
CriePir174	CRISPR array	AcrIF3
CriePir177	CRISPR array	AcrIF3
CriePir178	CRISPR-Cas I-E, III-U, I-F	AcrIF3
CriePir199	CRISPR-Cas I-E	AcrIE3
CriePir201	CRISPR array	AcrIF24, AcrIF23, AcrIF4
CriePir207	CRISPR-Cas III-U, I-F	AcrIF2, AcrIF3
CriePir249	CRISPR array	AcrIF3
CriePir274	CRISPR array	AcrIF3
CriePir311	CRISPR-Cas I-E, III-U, I-F	AcrIF3
CriePir318	CRISPR array	AcrIF3

**Table 2:** Significant correlations in the analyzed groups of CriePir *P. aeruginosa* isolates.

Correlation/Cas Type	No Cas	Single Cas	Double Cas	Triple Cas
Virulence genes vs. Antibiotic resistance genes	r = −0.61, *p* = 0.007	---	r = 0.58, *p* = 0.041	r = 0.9, *p* = 0.019
Active prophages vs. Antibiotic resistance genes	r = 0.8, *p* ˂ 0.0001	r = 0.576, *p* = 0.05	---	---
Active prophages vs. Virulence genes	r = −0.51, *p* = 0.03	r = −0.786, *p* = 0.003	---	---
Ambiguous prophages vs. Active prophages	---	---	r = −0.58, *p* = 0.040	r = −0.89, *p* = 0.013
CRISPR arrays vs. Antibiotic resistance genes	---	r = 0.63, *p* = 0.031	---	---
CRISPR arrays vs. Virulence genes	---	---	r = −0.6, *p* = 0.034	---
Ambiguous prophages vs. Anti-CRISPR proteins	---	---	---	r = −0.81, *p* = 0.048
Anti-CRISPR proteins vs. CRISPR4 arrays	---	r = −0.58, *p* = 0.031	---	---

## Data Availability

The bacterial genomes presented in this study are openly available in NCBI Genbank under the project number PRJNA744936.

## Supplementary Materials

The following are available online at https://www.mdpi.com/article/10.3390/antibiotics10111301/s1, Table S1. CriePir *P. aeruginosa* isolate typing and metadata, Table S2. List of reference *P. aeruginosa* isolates, Table S3. Reference isolates bearing Csx3 protein, Table S4. Antimicrobial resistance genotypes of CriePir *P. aeruginosa* isolates, Table S5. Plasmids found in clinical CriePir *P. aeruginosa* isolates from a Moscow multidisciplinary medical center, Table S6. The list of virulence genes detected in CriePir clinical P. aeruginosa isolates (VFDB, accessed on 20 March 2021), Table S7. Differences in virulence factor frequencies among the CriePir *P. aeruginosa* isolates with different CRISPR/Cas systems; Table S8. Results of MegaBLAST annotation of spacer sequences revealed in the genomes of CriePir P. aeruginosa isolates, Figure S1. The number of antibiotic resistance genes (**A**), plasmid number (**B**), and the number of genes encoding virulence factors (**C**) in the analyzed groups of the CriePir *P. aeruginosa* isolates, Figure S2. Phylogenetic trees for anti-CRISPR genes found in clinical CriePir *P. aeruginosa* isolates, Figure S3. Comparison of CriePir *P. aeruginosa* isolates with different CRISPR/Cas systems by the number of CRISPR arrays, Figure S4. Comparison of the number of active (**A**) and ambiguous (**B**) prophages between groups of CriePir *P. aeruginosa* isolates having either “CRISPR/No Cas”, “CRISPR/Single Cas”, “CRISPR/Double Cas” or “CRISPR/Triple Cas”, Figure S5. Comparison of the number of active and ambiguous prophages among CriePir *P. aeruginosa* isolates belonging to “CRISPR/Single Cas” (**A**), “CRISPR/Double Cas”, (**B**) or “CRISPR/Triple Cas” (**C**) groups, Figure S6. Correlation matrices for “CRISPR/No Cas”, “CRISPR/Single Cas”, “CRISPR/Double Cas”, and “CRISPR/Triple Cas” CriePir *P. aeruginosa* isolate data sets, Figure S7. CRISPR4 array length (**A**) and number of spacers (**B**) for CriePir *P. aeruginosa* isolates with Type I-F and Type I-E CRISPR/Cas systems, Figure S8. CriePir *P. aeruginosa* isolates’ spacer similarity.

## Abbreviations

Acr	anti-CRISPR
AR	antibiotic resistance
CC	clonal complex
CRISPR	clustered regularly interspaced short palindromic repeats
MDR	multidrug-resistant
MLST	multilocus sequence typing
OCL	Outer-core loci
ST	sequence type
UTI	urinary tract infections
