# Supplementary material for: CRISPR Element Patterns vs. Pathoadaptability of Clinical Pseudomonas aeruginosa Isolates from a Medical Center in Moscow, Russia
Source: Antibiotics (Basel). 2021 Oct 26;10(11):1301. doi: 10.3390/antibiotics10111301 (PMC8615150; doi:10.3390/antibiotics10111301)
Supplement: Supplementary file 1 [file antibiotics-10-01301-s001.zip › suppl_figures.pdf]

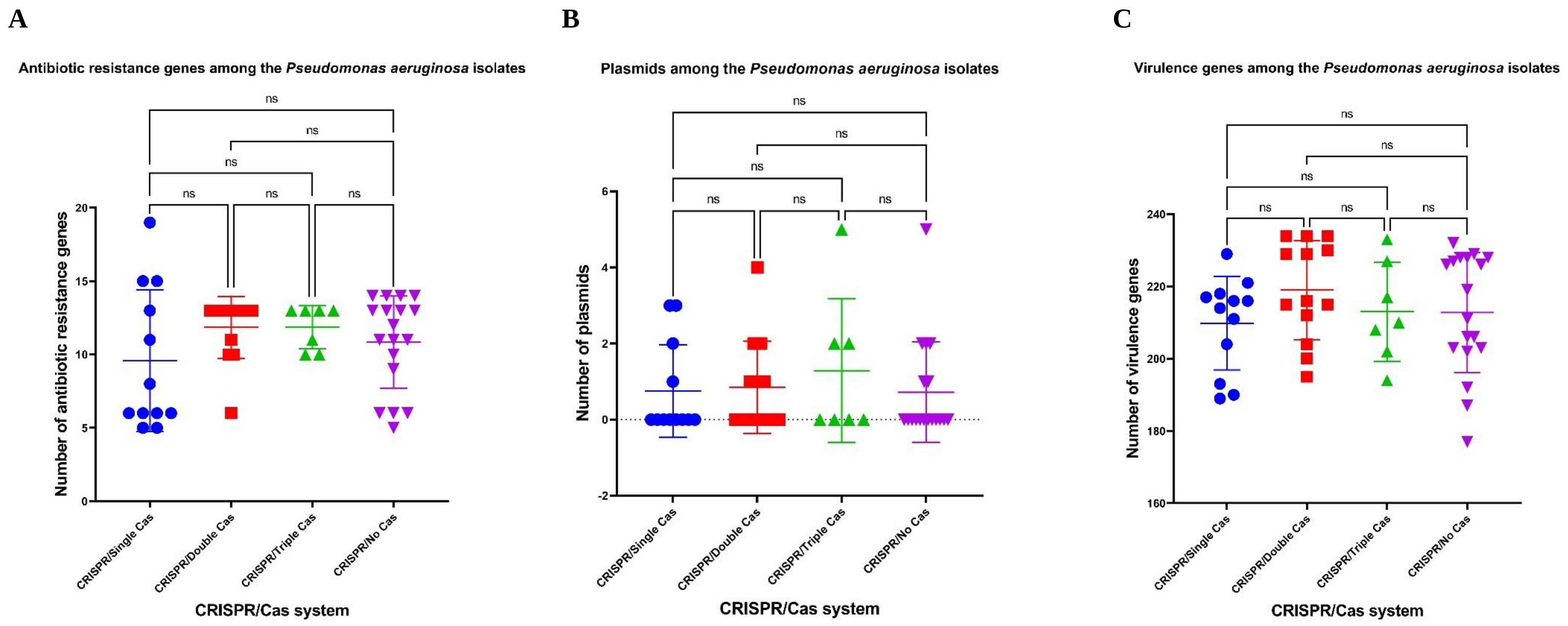

Figure S1. The number of antibiotic resistance genes (A), plasmid number (B) and the number of genes encoding virulence factors (C) in the analyzed groups of the CriePir *P. aeruginosa* isolates

Figure S2. Phylogenetic trees for anti-CRISPR genes found in clinical CriePir *P. aeruginosa* isolates

S2.1. AcrIE3

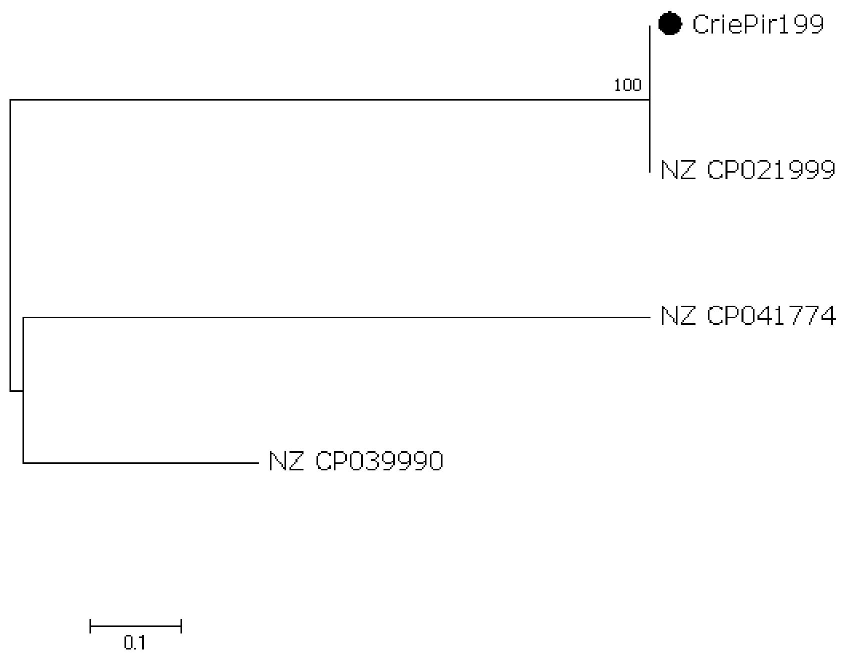

S2.2. AcrIF2

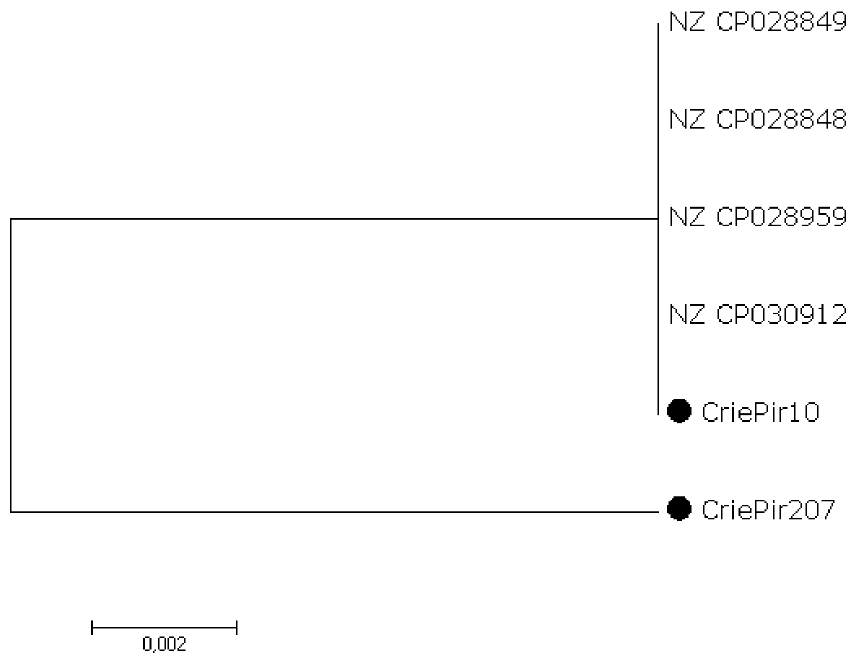

S2.3. AcrIF3

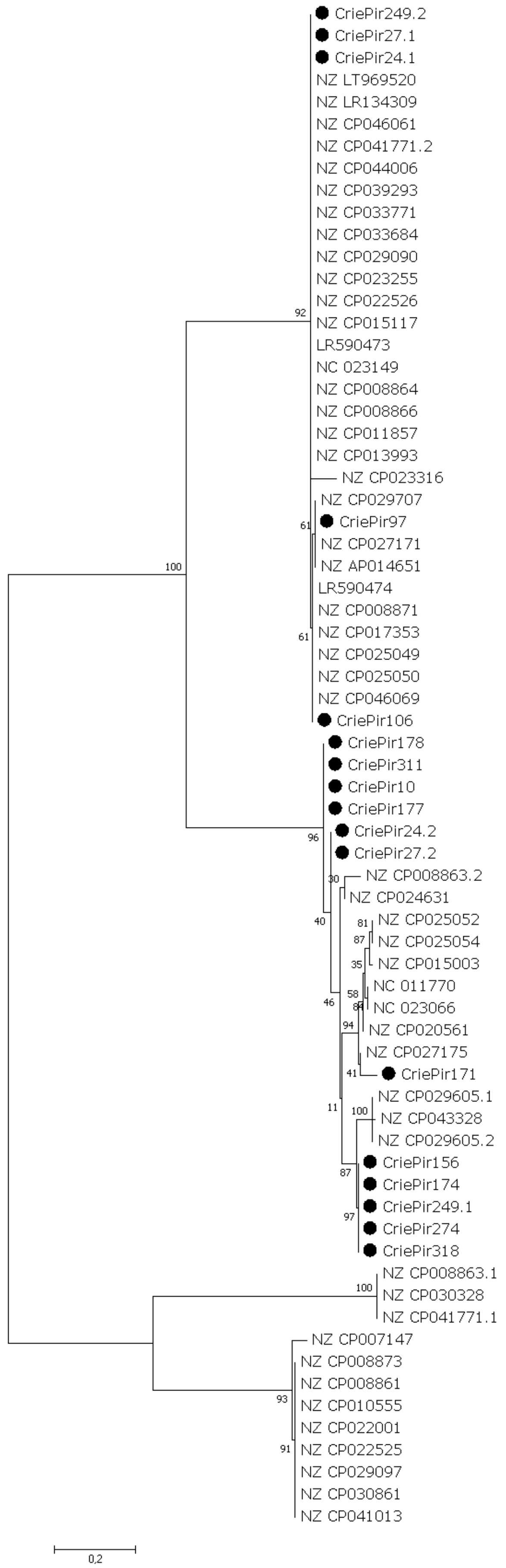
CriePir249.2
CriePir27.1
CriePir24.1
NZ LT969520
NZ LR134309
NZ CP046061
NZ CP041771.2
NZ CP044006
NZ CP039293
NZ CP033771
NZ CP033684
NZ CP029090
NZ CP023255
NZ CP022526
NZ CP015117
LR590473
NC 023149
NZ CP008864
NZ CP008866
NZ CP011857
NZ CP013993
NZ CP023316
NZ CP029707
CriePir97
NZ CP027171
NZ AP014651
LR590474
NZ CP008871
NZ CP017353
NZ CP025049
NZ CP025050
NZ CP046069
CriePir106
CriePir178
CriePir311
CriePir10
CriePir177
CriePir24.2
CriePir27.2
NZ CP008863.2
NZ CP024631
NZ CP025052
NZ CP025054
NZ CP015003
NC 011770
NC 023066
NZ CP020561
NZ CP027175
CriePir171
NZ CP029605.1
NZ CP043328
NZ CP029605.2
CriePir156
CriePir174
CriePir249.1
CriePir274
CriePir318
NZ CP008863.1
NZ CP030328
NZ CP041771.1
NZ CP007147
NZ CP008873
NZ CP008861
NZ CP010555
NZ CP022001
NZ CP022525
NZ CP029097
NZ CP030861
NZ CP041013
0.2

S2.4. AcrIF4

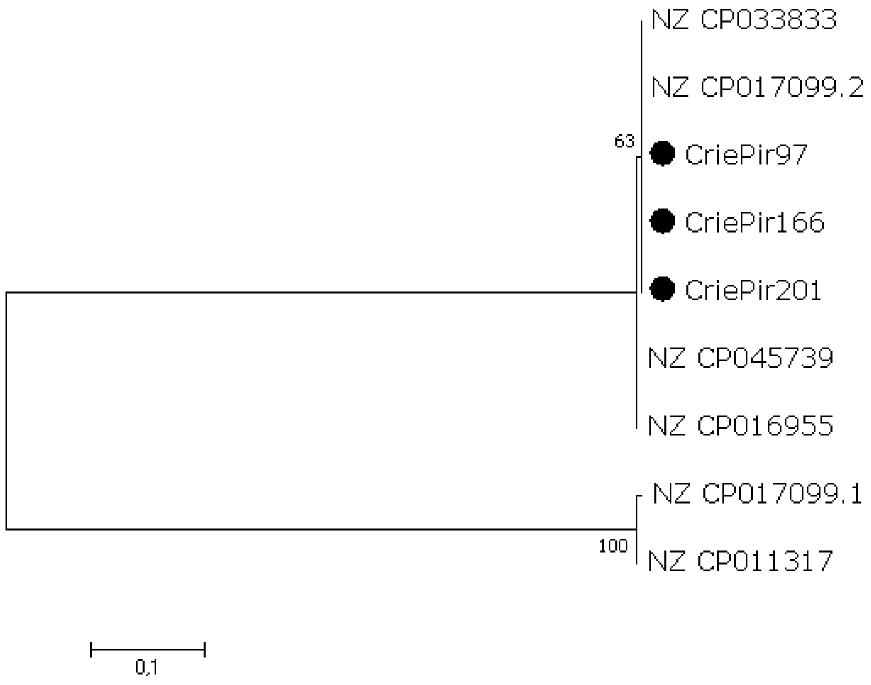
NZ CP033833
NZ CP017099.2
63
CriePir97
CriePir166
CriePir201
NZ CP045739
NZ CP016955
NZ CP017099.1
100
NZ CP011317
0,1

S2.5. AcrIIC2

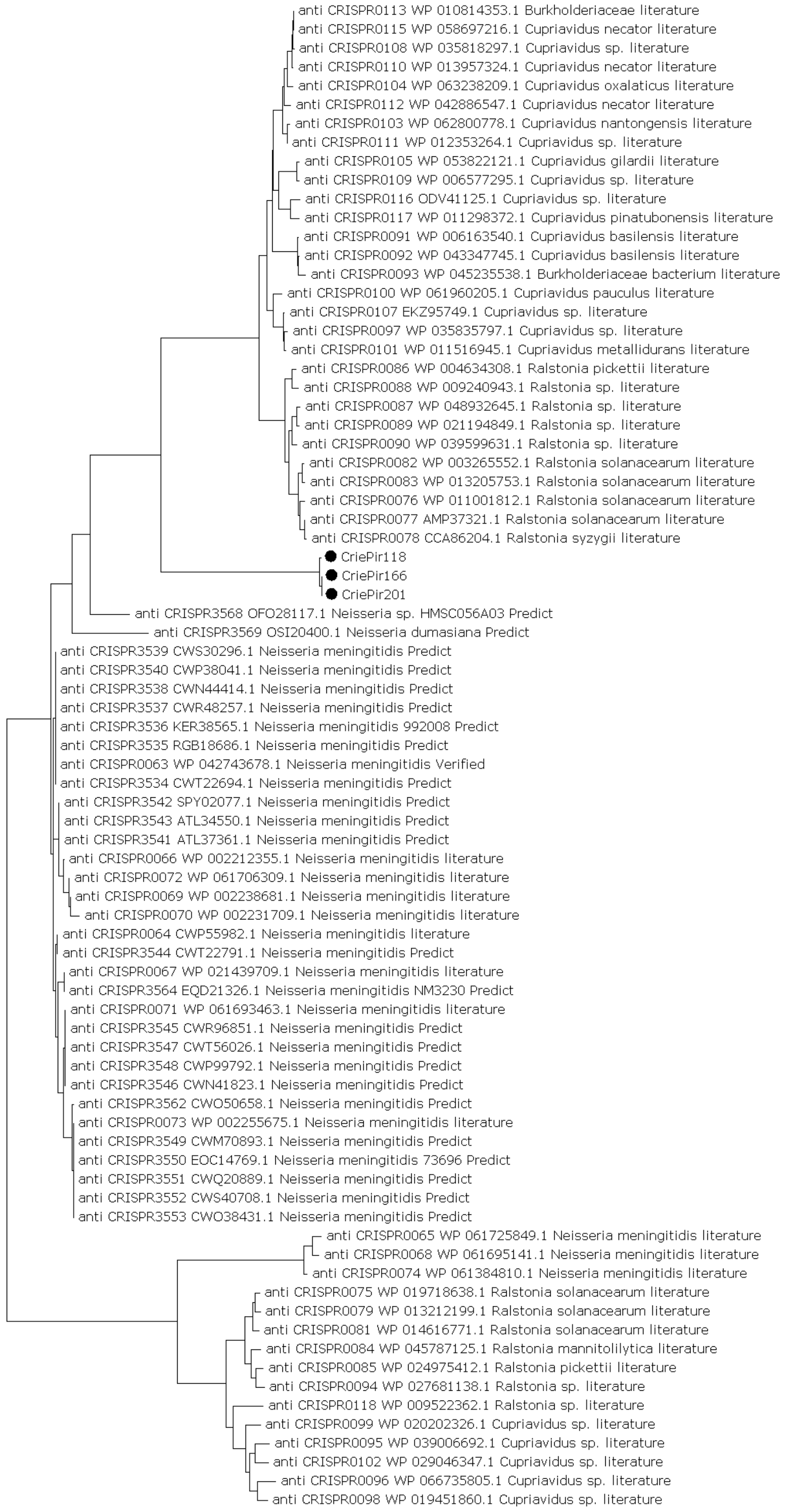
anti CRISPR0113 WP 010814353.1 Burkholderiaceae literature
anti CRISPR0115 WP 058697216.1 Cupriavidus necator literature
anti CRISPR0108 WP 035818297.1 Cupriavidus sp. literature
anti CRISPR0110 WP 013957324.1 Cupriavidus necator literature
anti CRISPR0104 WP 063238209.1 Cupriavidus oxalaticus literature
anti CRISPR0112 WP 042886547.1 Cupriavidus necator literature
anti CRISPR0103 WP 062800778.1 Cupriavidus nantongensis literature
anti CRISPR0111 WP 012353264.1 Cupriavidus sp. literature
anti CRISPR0105 WP 053822121.1 Cupriavidus gilardii literature
anti CRISPR0109 WP 006577295.1 Cupriavidus sp. literature
anti CRISPR0116 ODV41125.1 Cupriavidus sp. literature
anti CRISPR0117 WP 011298372.1 Cupriavidus pinatubonensis literature
anti CRISPR0091 WP 006163540.1 Cupriavidus basilensis literature
anti CRISPR0092 WP 043347745.1 Cupriavidus basilensis literature
anti CRISPR0093 WP 045235538.1 Burkholderiaceae bacterium literature
anti CRISPR0100 WP 061960205.1 Cupriavidus pauculus literature
anti CRISPR0107 EKZ95749.1 Cupriavidus sp. literature
anti CRISPR0097 WP 035835797.1 Cupriavidus sp. literature
anti CRISPR0101 WP 011516945.1 Cupriavidus metallidurans literature
anti CRISPR0086 WP 004634308.1 Ralstonia pickettii literature
anti CRISPR0088 WP 009240943.1 Ralstonia sp. literature
anti CRISPR0087 WP 048932645.1 Ralstonia sp. literature
anti CRISPR0089 WP 021194849.1 Ralstonia sp. literature
anti CRISPR0090 WP 039599631.1 Ralstonia sp. literature
anti CRISPR0082 WP 003265552.1 Ralstonia solanacearum literature
anti CRISPR0083 WP 013205753.1 Ralstonia solanacearum literature
anti CRISPR0076 WP 011001812.1 Ralstonia solanacearum literature
anti CRISPR0077 AMP37321.1 Ralstonia solanacearum literature
anti CRISPR0078 CCA86204.1 Ralstonia syzygii literature
CriePir118
CriePir166
CriePir201
anti CRISPR3568 OFO28117.1 Neisseria sp. HMSC056A03 Predict
anti CRISPR3569 OSI20400.1 Neisseria dumasiana Predict
anti CRISPR3539 CWS30296.1 Neisseria meningitidis Predict
anti CRISPR3540 CWP38041.1 Neisseria meningitidis Predict
anti CRISPR3538 CWN44414.1 Neisseria meningitidis Predict
anti CRISPR3537 CWR48257.1 Neisseria meningitidis Predict
anti CRISPR3536 KER38565.1 Neisseria meningitidis 992008 Predict
anti CRISPR3535 RGB18686.1 Neisseria meningitidis Predict
anti CRISPR0063 WP 042743678.1 Neisseria meningitidis Verified
anti CRISPR3534 CWT22694.1 Neisseria meningitidis Predict
anti CRISPR3542 SPY02077.1 Neisseria meningitidis Predict
anti CRISPR3543 ATL34550.1 Neisseria meningitidis Predict
anti CRISPR3541 ATL37361.1 Neisseria meningitidis Predict
anti CRISPR0066 WP 002212355.1 Neisseria meningitidis literature
anti CRISPR0072 WP 061706309.1 Neisseria meningitidis literature
anti CRISPR0069 WP 002238681.1 Neisseria meningitidis literature
anti CRISPR0070 WP 002231709.1 Neisseria meningitidis literature
anti CRISPR0064 CWP55982.1 Neisseria meningitidis literature
anti CRISPR3544 CWT22791.1 Neisseria meningitidis Predict
anti CRISPR0067 WP 021439709.1 Neisseria meningitidis literature
anti CRISPR3564 EQD21326.1 Neisseria meningitidis NM3230 Predict
anti CRISPR0071 WP 061693463.1 Neisseria meningitidis literature
anti CRISPR3545 CWR96851.1 Neisseria meningitidis Predict
anti CRISPR3547 CWT56026.1 Neisseria meningitidis Predict
anti CRISPR3548 CWP99792.1 Neisseria meningitidis Predict
anti CRISPR3546 CWN41823.1 Neisseria meningitidis Predict
anti CRISPR3562 CWO50658.1 Neisseria meningitidis Predict
anti CRISPR0073 WP 002255675.1 Neisseria meningitidis literature
anti CRISPR3549 CWM70893.1 Neisseria meningitidis Predict
anti CRISPR3550 EOC14769.1 Neisseria meningitidis 73696 Predict
anti CRISPR3551 CWQ20889.1 Neisseria meningitidis Predict
anti CRISPR3552 CWS40708.1 Neisseria meningitidis Predict
anti CRISPR3553 CWO38431.1 Neisseria meningitidis Predict
anti CRISPR0065 WP 061725849.1 Neisseria meningitidis literature
anti CRISPR0068 WP 061695141.1 Neisseria meningitidis literature
anti CRISPR0074 WP 061384810.1 Neisseria meningitidis literature
anti CRISPR0075 WP 019718638.1 Ralstonia solanacearum literature
anti CRISPR0079 WP 013212199.1 Ralstonia solanacearum literature
anti CRISPR0081 WP 014616771.1 Ralstonia solanacearum literature
anti CRISPR0084 WP 045787125.1 Ralstonia mannitolilytica literature
anti CRISPR0085 WP 024975412.1 Ralstonia pickettii literature
anti CRISPR0094 WP 027681138.1 Ralstonia sp. literature
anti CRISPR0118 WP 009522362.1 Ralstonia sp. literature
anti CRISPR0099 WP 020202326.1 Cupriavidus sp. literature
anti CRISPR0095 WP 039006692.1 Cupriavidus sp. literature
anti CRISPR0102 WP 029046347.1 Cupriavidus sp. literature
anti CRISPR0096 WP 066735805.1 Cupriavidus sp. literature
anti CRISPR0098 WP 019451860.1 Cupriavidus sp. literature

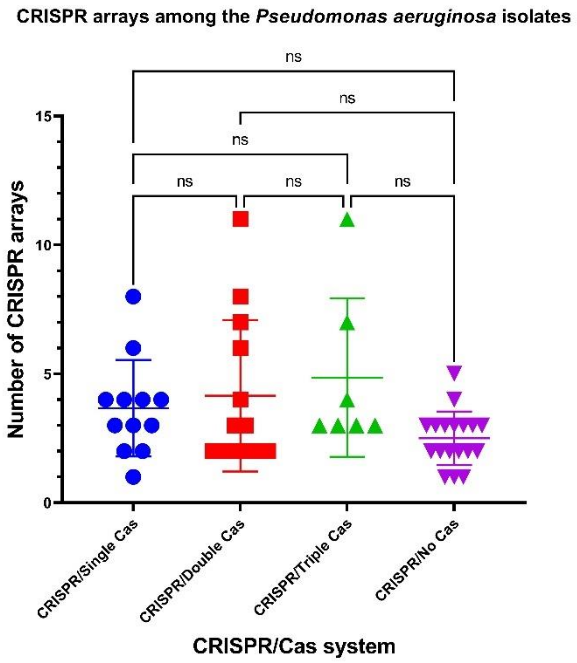

Figure S3. Comparison of CriePir *P. aeruginosa* isolates having different CRISPR/Cas systems by the number of CRISPR arrays

A

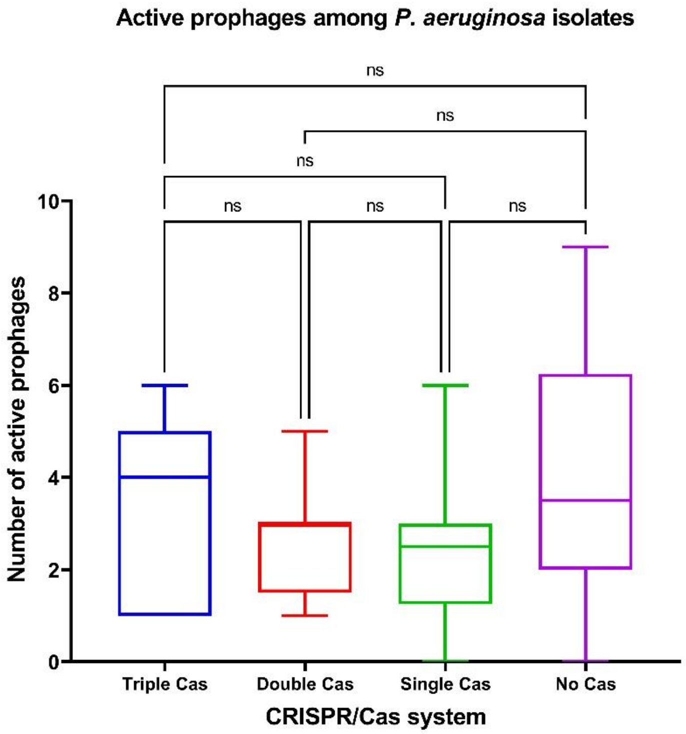

B

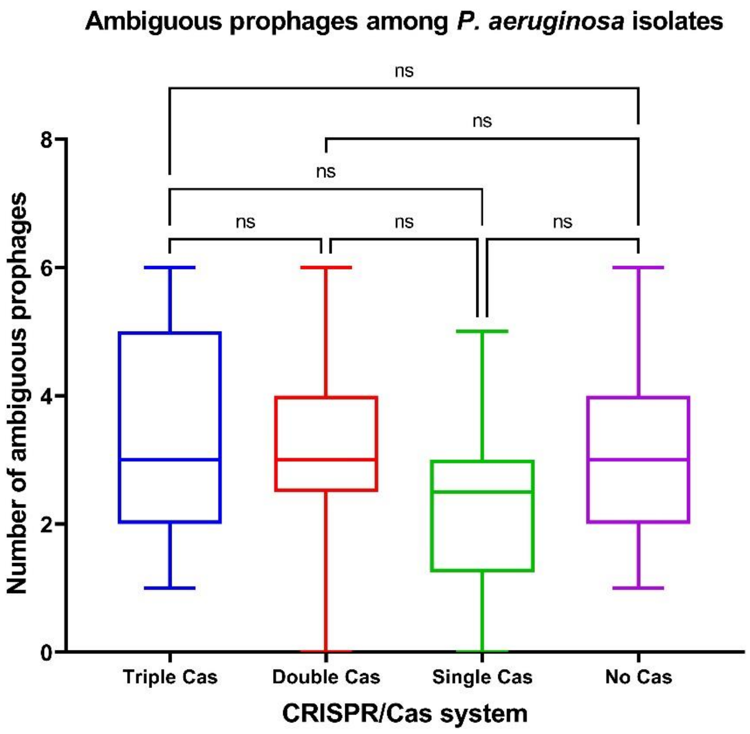

Figure S4. Comparison of the number of active (A) and ambiguous (B) prophages between groups of CriePir *P. aeruginosa* isolates having either "CRISPR/No Cas", "CRISPR/Single Cas", "CRISPR/Double Cas" or "CRISPR/Triple Cas"

A

B

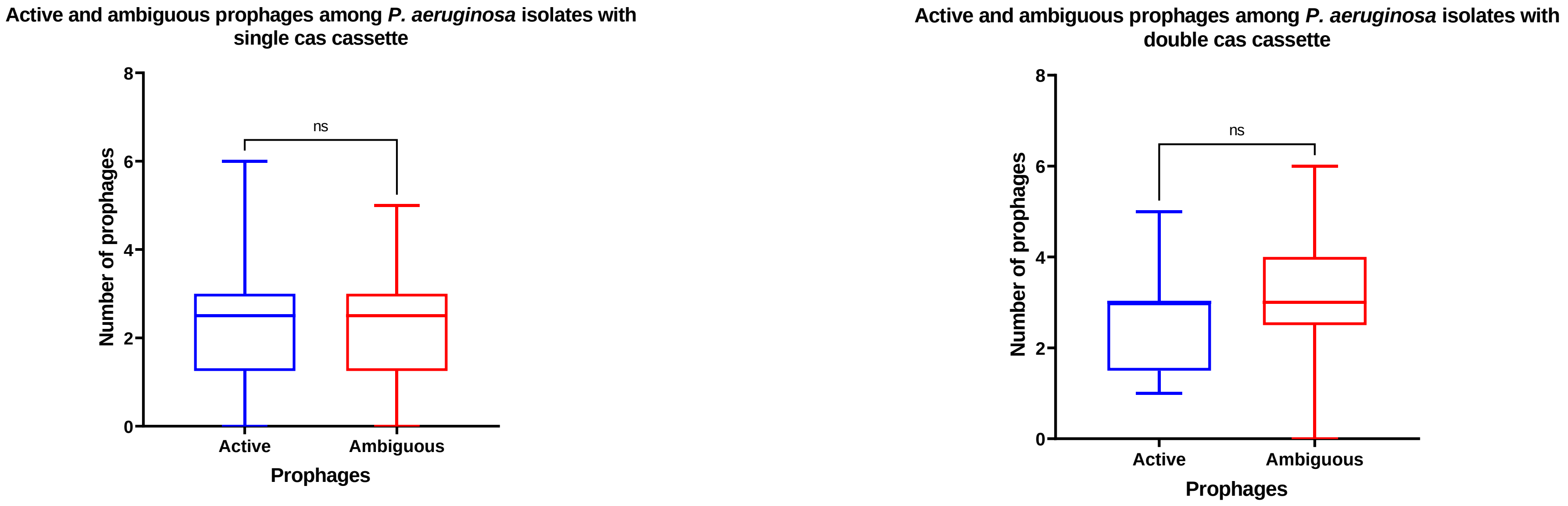

C

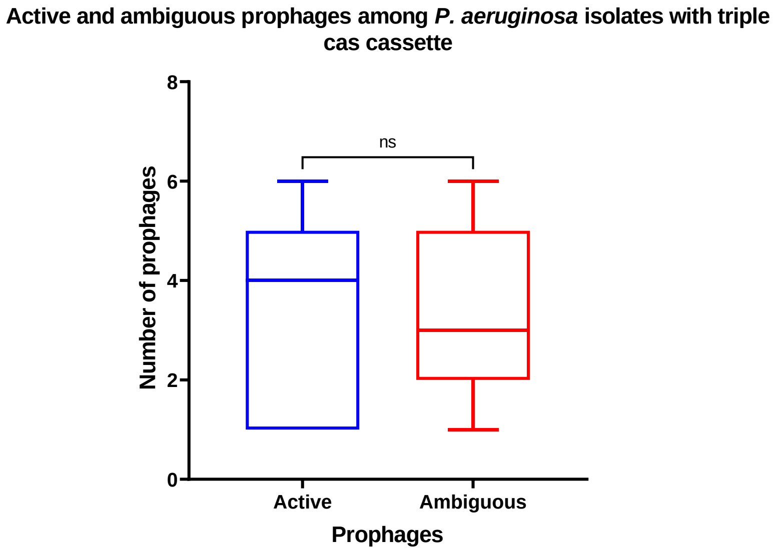

Figure S5. Comparison of the number of active and ambiguous prophages among CriePir *P. aeruginosa* isolates belonging to “CRISPR/Single Cas” (A), “CRISPR/Double Cas” (B) or “CRISPR/Triple Cas” (C) groups

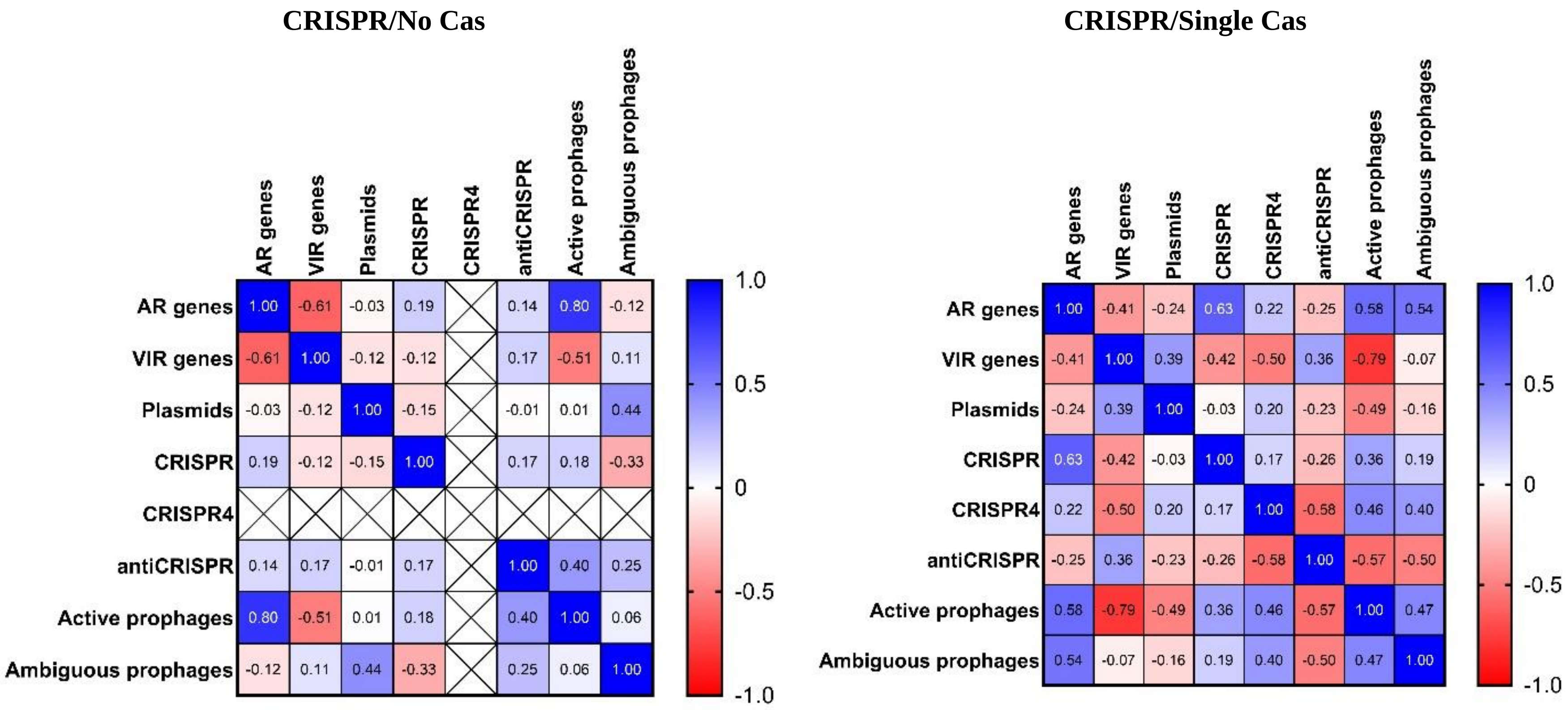

Figure S6. Correlation matrices for “CRISPR/No Cas”, “CRISPR/Single Cas”, “CRISPR/Double Cas” and “CRISPR/Triple Cas” CriePir *P. aeruginosa* isolate data sets

CRISPR/Double Cas

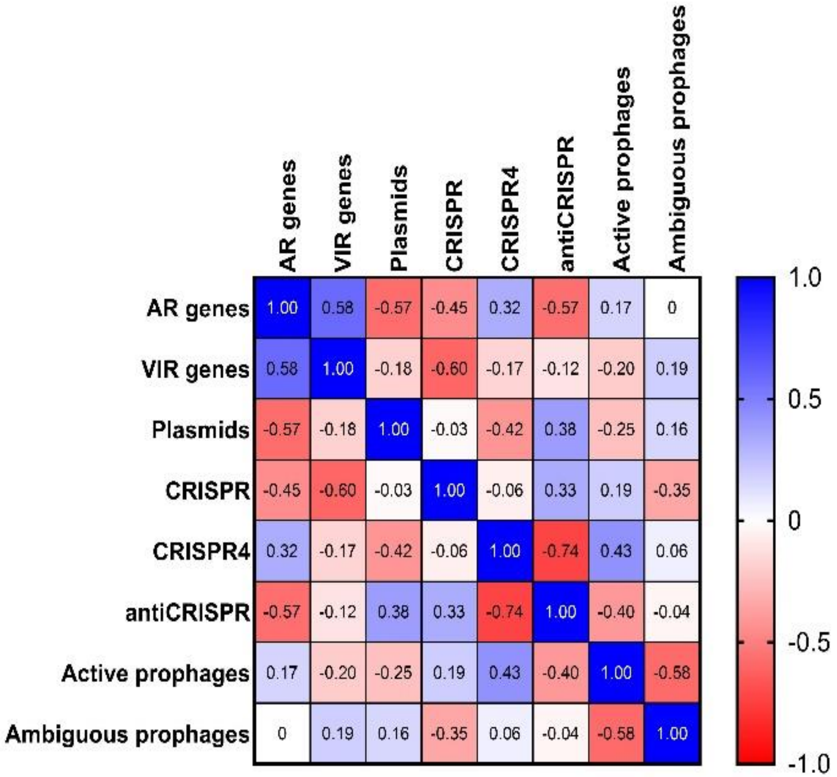

CRISPR/Triple Cas

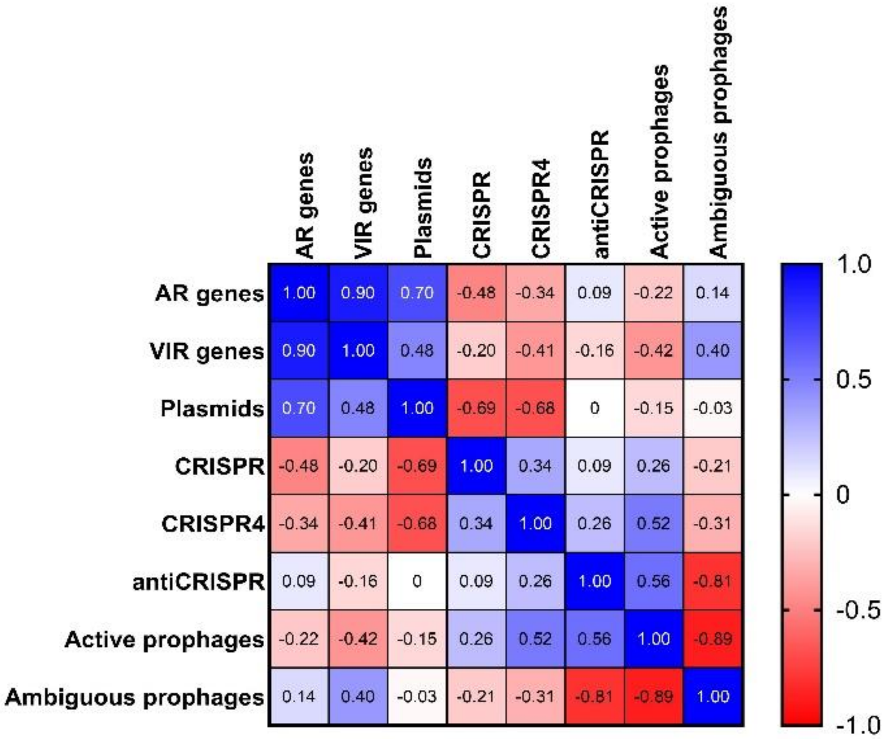

Figure S6 (continued).

A

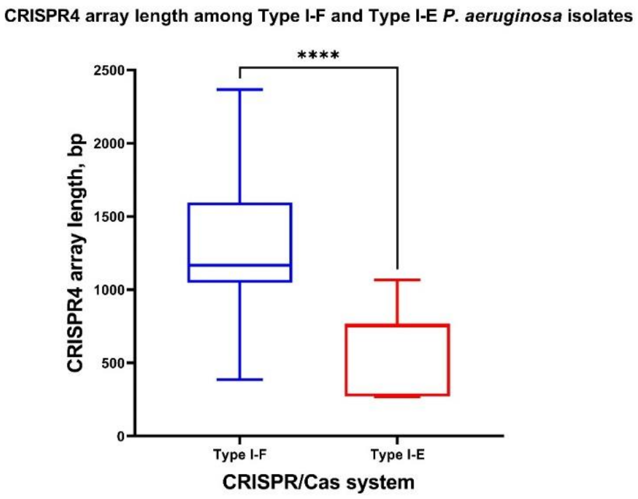

B

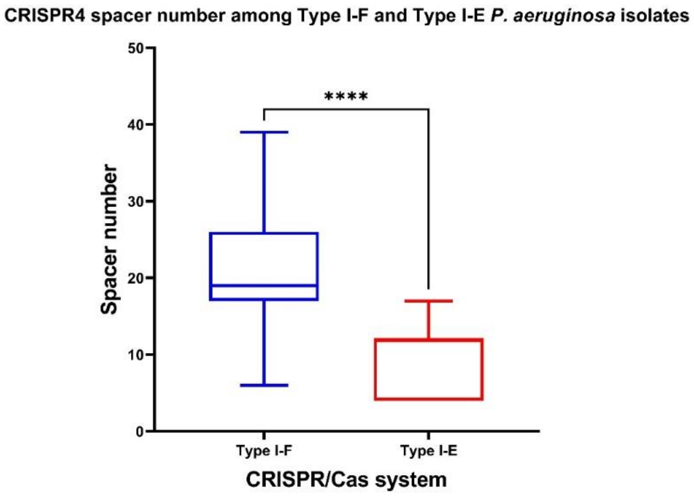

Figure S7. CRISPR4 array length (A) and number of spacers (B) for CriePir *P. aeruginosa* isolates with Type I-F and Type I-E CRISPR/Cas systems

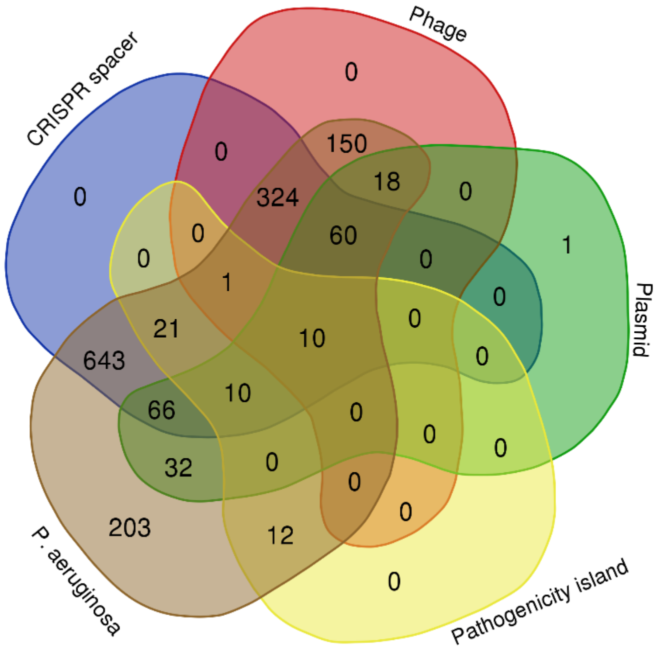

Figure S8. CriePir *P. aeruginosa* isolates' spacer similarity
